# Large scale uniform Ni-P plated carbon fiber for boosting urea electro-oxidation and electro-detection

**DOI:** 10.3389/fchem.2023.1298655

**Published:** 2023-10-26

**Authors:** Yan-Ru Fan, Jin-Qi Li, Yu-Xi Yang, Zhi-Hao Zhang, Jie Zhang, Jing-He Yang

**Affiliations:** ^1^ Clinical Lab Department, Henan Provincial People’s Hospital, Zhengzhou, China; ^2^ School of Chemistry Engineering, Zhengzhou University, Zhengzhou, China; ^3^ School of Ecology and Environment, Zhengzhou University, Zhengzhou, China; ^4^ Department of Infections Disease, The First Affiliated Hospital of Zhengzhou University, Zhengzhou, China

**Keywords:** urea oxidation, carbon fibers, electroless plating, electrocatalyst, urea detection

## Abstract

Seeking an excellent electrocatalyst is the trickiest issue for the application of urea electro-oxidation and electro-detection. Phosphorus-doped nickel plating on carbon fibers (Ni-P/CF) is synthesized by simple electroless plating. SEM results exhibit that the Ni-P densely and uniformly grows onto the surface of carbon fibers (CF), forming carbon fibers-like nanoarchitectures. Benefiting from the carbon fibers-like nano architectures with abundant exposed active sites on the surface of CF, electron transfer can be synchronously facilitated, and Ni-P/CF displays superior urea electrooxidation (UOR) performance with potentials of 1.40 V to reach 100 mA cm^−2^. Impressively, it can maintain at 20 mA cm^−2^ for 48 h without evident activity attenuation, demonstrating robust durability. Cycle stability shows that the voltage has only increased by 10 mV at 300 mA cm^−2^ from the 10th to 20000th cycles. Most importantly, Ni-P/CF at a length of 100 cm with good reproducibility was successfully synthesized, denoting great potential for large-scale industrial production. Therefore, this work not only affords cost-effective tactics for urea-rich wastewater degradation but also can achieve practical medical applications.

## 1 Introduction

Urea is the end product of amino acid catabolism in the body and the main component of non-protein nitrogen in the blood. Urea is mainly excreted by the kidneys, all urea in the blood is filtered by the glomerulus, 30%–40% is reabsorbed by the renal tubules, and the renal tubules also secrete a small amount of urea, and the secretion increases in severe renal failure. Measurement of serum urea is one of the commonly used measures of glomerular function. Besides, urea has recently emerged as a bridging molecule connecting water and energy. The application of urea detection mainly has aspects in the feed industry, chemical industry, agriculture, and environmental protection industry. As a prominent and effective hydrogen carrier, urea is relatively non-toxic and non-flammable. Most importantly, the manufacturing infrastructure of cheap and widely available urea has developed, and the output is increasing rapidly (King and Botte, 2011; Sun et al., 2020; Li et al., 2021; Safeer N et al., 2022). However, in actual production and life, the discharged directly of urea-containing wastewater from industry, animal husbandry, and agriculture will cause serious environmental problems and even threaten human health (Urbańczyk et al., 2016; Lu et al., 2019; Sun and Ding, 2020). At present, the treatment methods of urea-rich wastewater mainly include hydrolysis (Mahalik et al., 2010), enzymatic hydrolysis (Krajewska, 2009), adsorption (Xue and Wilson, 2016), electrochemical oxidation, *etc.* Among them, electrochemical oxidation has gradually become one of the most potent methods because of its convenience for subsequent treatment and utilization, less waste (Guo et al., 2016; Xu et al., 2021; Zaher and Shehata, 2021). Urea electrolysis combines wastewater remediation and energy conversion, which provides a promising method to solve the increasingly severe environmental and fuel crisis. In addition, the theoretical oxidation potential of urea oxidation reaction (UOR) is only 0.37 V, which can effectively reduce the output of hydrogen production energy (Xiao et al., 2017; Yang et al., 2019; Wu and Hou, 2021). However, its characteristic of slow initiation kinetics of the six-electron transfer process (CO(NH_2_)_2_ (aq) + 6OH^−^ → N_2_ (g) + CO_2_ (g) + 5H_2_O (l) + 6e^−^) hinders the overall electrolysis efficiency. Therefore, seeking an excellent electrocatalyst is the trickiest issue for the application of urea electrooxidation (Tong et al., 2018; Yang et al., 2019; Zhu et al., 2020). Precious metals such as platinum, rhodium, and iridium have shown fantastic electrocatalytic activity in previous reports, but they are difficult to apply in practical industries owing to their exorbitant price, and scarcity (Amstutz et al., 2012; Bai et al., 2020; Camposeco et al., 2021; Wang et al., 2021). Therefore, finding alternative and inexpensive non-noble metal catalysts is a critical factor to solving the problem. Transition metals are stable in an electrolyte solution and their crustal reserves are abundant, which has become popular research for the past few years (Liu et al., 2020; Feng et al., 2020; Zhang et al., 2020). At present, urea detection mainly has the following methods. The urease method is currently the most commonly used urea detection method, and its principle is to use urease to catalyze urea hydrolysis to generate ammonia, and then use acid-base titration or electrochemical method to determine the content of ammonia. Color rendering is another commonly used urea detection method, the principle of which is to condense urea with diacetyl to form diazine with color, and then quantify according to the depth of color. Fluorescence method is a urea detection method developed in recent years, the principle of which is to use urea to react with specific reagents to generate fluorescent substances, and then quantify according to the fluorescence intensity. With the continuous development of science and technology, the future urea detection technology will develop in a more sensitive, accurate and intelligent direction.

According to the research on the catalysis of urease, nickel is the active site for the dissociation of urea molecules (Suárez et al., 2003; Krajewska, 2009; Yan et al., 2012; Sha et al., 2020a). In recent years, Ni-based catalysts containing heteroatoms (N, P, S) with rich active sites, unique molecular structure, and robust electrochemical activity and stability have become the focus of studying (Sha et al., 2020b; Hu et al., 2020; Wang et al., 2022). Controlling the nanostructure of the electrode by carbon coating can effectively improve the active area and electrical conductivity of the material. The fabrication of the Ni_2_P-C catalyst was done by Yang et al. using a simple hydrothermal method. In the alkaline electrolyte, the Ni_2_P-C catalyst only needs potential of 0.5 V to reach the current density of 70.4 mA cm^-2^ (Zhang et al., 2017). In addition, hierarchical nanostructures may afford more accessible active sites and high durability in electrolytes. Li et al. (2020) obtained a uniform porous rod-like Ni_2_P/Ni composite via a simple phosphating method. The DFT calculation results showed that Ni_2_P/Ni fully embodies the size impact of nanomaterials, which makes the surface of the material easier to activate and significantly improves the kinetics and conductivity for UOR. Carbon fibers (CFs) with high strength and high modulus have a wide range of applications, including sensors, composite reinforcement materials, general engineering, and aerospace. However, the resistivity of carbon fibers is relatively large, and surface metallization technology including ion plating, metal powder plating, electroless plating, and electroplating can effectively improve this situation (Wang et al., 2017; Liu et al., 2019). Among them, electroless plating is an autocatalytic method widely used in industry. In the absence of electricity, the substrate is immersed in an electroless bath mainly composed of metal salts, stabilizers, reducing agents, and complexing agents to generate potential charges. The metals can be continuously and uniformly deposited on the substrate, effectively enhancing the thermal conductivity and electrical conductivity of the substrate. Temperature, pH value, bath parameters and so on directly affect the electroless plating process (Fayyad et al., 2018; Choi et al., 2020). In addition, electroless plating is simple to operate and results in good surface deposition with uniform covering, which is suitable for all kinds of non-conductive substrates with complex shapes. Most importantly, electroless plating with low cost and mild experimental conditions makes it possible for large-scale industrial production and exhibits nonnegligible competitiveness (Zheng et al., 2019; Qi et al., 2020).

**SCHEME 1:**
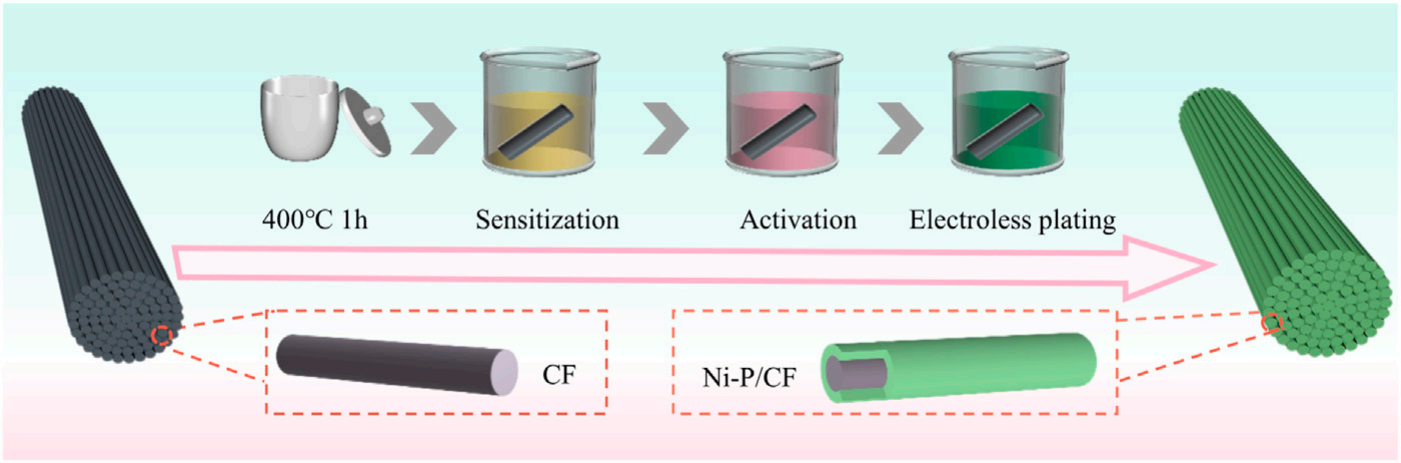
Schematic diagram of synthesis route of Ni-P/CF.

In this study, we metalized the surface of carbon fibers by electroless nickel plating to obtain Ni-P/CF, which showed outstanding catalytic performance for urea electrooxidation (as exhibited in Scheme 1). Further, we explored the optimal conditions for electroless nickel plating on carbon fibers by controlling the heat treatment temperature, Ni/P ratio, and pH value of the plating solution. The experimental result display that the optimal treatment temperature of the plating solution is 80°C, the optimal Ni/P ratio is 1.5:2, and the optimal pH value is 8.5. In addition, carbon cloth and carbon paper were also electroless nickel plated as comparative substrates. It is worth mentioning that the low-cost and easy-to-operate electroless nickel plating process is of great importance for achieving large-scale production. As a demonstration, we successfully synthesized nickel-plated carbon fibers with a length of 100 cm in the laboratory, which undoubtedly provided infinite possibilities for practical industrial and medical application.

## 2 Experimental section

### 2.1 Experimental reagents

Nickel chloride (NiCl_2_•6H_2_O) and Sodium hypophosphite monohydrate (NaH_2_PO_2_•H_2_O) were purchased from Shanghai Macklin Biochemical Co., Ltd. Trisodium citrate dihydrate (Na_3_C_6_H_5_O_7_•2H_2_O), Ammonium chloride (NH_4_Cl), Palladium chloride (PdCl_2_), potassium hydroxide (KOH) and urea (CH_4_N_2_O) were purchased from Sinopharm Chemical Reagent Co., Ltd. Tin(Ⅱ) chloride dihydrate (SnCl_2_•2H_2_O) was purchased from Tianjin Beichen District Fangzheng Reagent Factory. All the reagents of this work were used without further purification.

### 2.2 Pretreatment of carbon fibers

Firstly, the carbon fibers were placed in a muffle furnace at 400°C for 1 h to remove organic impurities and pollutants on the surface of the carbon fibers, followed by sensitization and activation treatment: the carbon fibers were immersed in 0.05 M SnCl_2_ containing 0.15 M HCl for 30 min to make Sn^2+^ adhere to the surface of the carbon fibers. After that, the carbon fiber was transferred to 0.6 mM PdCl_2_ containing 0.01 M HCl solution for activation for 30 min. In this process, the sensitized substance is further oxidized to form a thin layer of catalytic metal on the surface of the carbon fiber, which acts as a catalyst for the redox reaction during electroless plating. Finally, cleaned with acetone, ethanol, and water by ultrasonic for 15 min, and then dried in an oven at 60°C.

### 2.3 Pretreatment of carbon cloth and carbon paper

The carbon cloth and carbon paper as a comparison were treated with oil and impurity removal by reference (Li S. et al., 2021; Gao et al., 2021), respectively. The carbon cloth of 1 cm × 1 cm was ultrasonicated for 20 min in a mixed solution (10 wt% nitric acid and 10 wt% sulfuric acid, V_nitric acid_: V_sulfuric acid_ = 3:1), then cleaned with ethanol and deionized water successively. Finally, the carbon cloth was dried at 60°C for 12 h. The carbon paper was cut into 1 cm × 1 cm and ultrasonic cleaned with acetone, ethanol, and water to remove dust or grease from the surface. Subsequently, the carbon paper was soaked in 10 mL 20 mM nitric acid for ultrasonic treatment for 10 min, then transferred to an autoclave lined with Teflon. It was heated at 120°C for 12 h. Finally, the carbon paper was washed with water and dried for storage.

The subsequent sensitization and activation processes of carbon cloth and carbon paper were the same as that of carbon fibers.

### 2.4 Synthesis of Ni-P/CF

The pre-treated CFs (2 cm) were immersed in an electroless bath composed of NiCl_2_ ·6H_2_O (3.75 mmol), NaH_2_PO_2_ (5 mmol), Na_3_C_6_H_5_O_7_ 2H_2_O (1.75 mmol), NH_4_Cl (7.5 mmol), and water (25 mL). The pH of the electroless plating solution was adjusted to 8.5 by ammonia. Then the beaker was transferred to the oven at 80°C for 1 h. After electroless plating, carbon fibers were washed thoroughly with ethanol and water and then dried at 60°C to obtain Ni-P/CF-80. Ni-P/CF-60 and Ni-P/CF-100 were prepared with the same synthesis procedure as Ni-P/CF-80 except that the heat treatment temperature was 80°C and 100°C, respectively. In addition, the pH and Ni/P ratio of the bath were also investigated. The synthesis procedure of Ni-P/CF pH_plating aq._ = 8 and Ni-P/CF pH_plating aq._ = 9 were the same as those of Ni-P/CF-80 except that the pH was 8 and 9, respectively. The synthesis procedure of Ni-P/CF 1:1 and Ni-P/CF 2:1.5 were the same as those of Ni-P/CF-80 except that The Ni/P ratio was 1:1 and 2:1.5, respectively.

### 2.5 Synthesis of Ni-P/CC and Ni-P/CP

Electroless nickel plating was also carried out on carbon cloth and carbon paper with the same synthesis procedure of Ni-P/CF-80.

### 2.6 Material characterization

The morphology and microstructure of the samples were observed by scanning electron microscopy (SEM, Sigma 300) combined with EDX (Smart EDX) measurement. The crystal structure and crystal plane information of the synthesized material were measured by X-ray diffraction (XRD, Bruker AXS D8 Advanced) in the scanning range of 10°–90° equipped with monochromatic Cu-Kα radiation (λ = 1.54 Å) at 40 mA and 45 kV. X-ray photoelectron spectroscopy (XPS) was measured by KRATOS (Axis Ultra^DLD^) spectrometer. The excitation source is Al ka ray (hv = 1,486.6 eV), and the binding energy of C1s = 284.80 eV is used as the energy standard for charge correction.

### 2.7 Electrochemical measurements

Electrochemical measurements were performed with a set of traditional three-electrode configurations consisting of Hg/HgO (1 M KOH) as the reference electrode, a carbon rod as a counter electrode, and the prepared Ni-P/CF as the working electrode. And the aqueous solution of 1 M KOH with and without 0.33 M urea was employed as the electrolyte for the measurements at room temperature. The obtained potentials were exhibited vs. RHE reference electrode in this work by the equation: E_RHE_ = E_Hg/HgO_ + 0.591 * pH + 0.098. The electrochemical properties of the materials were investigated by cyclic voltammetry (CV) at 50 mV s^-1^ with a potential ranging from 0.92 to 1.72 V (vs. RHE). Electrochemical impedance spectroscopy (EIS) was measured under the frequency range 0.01 Hz–100 KHz. The electrochemical stability was investigated using chronoamperometry and potential multistep chronopotentiometry measurement.

## 3 Results and discussion

### 3.1 Material characterization and discussion

**FIGURE 1:**
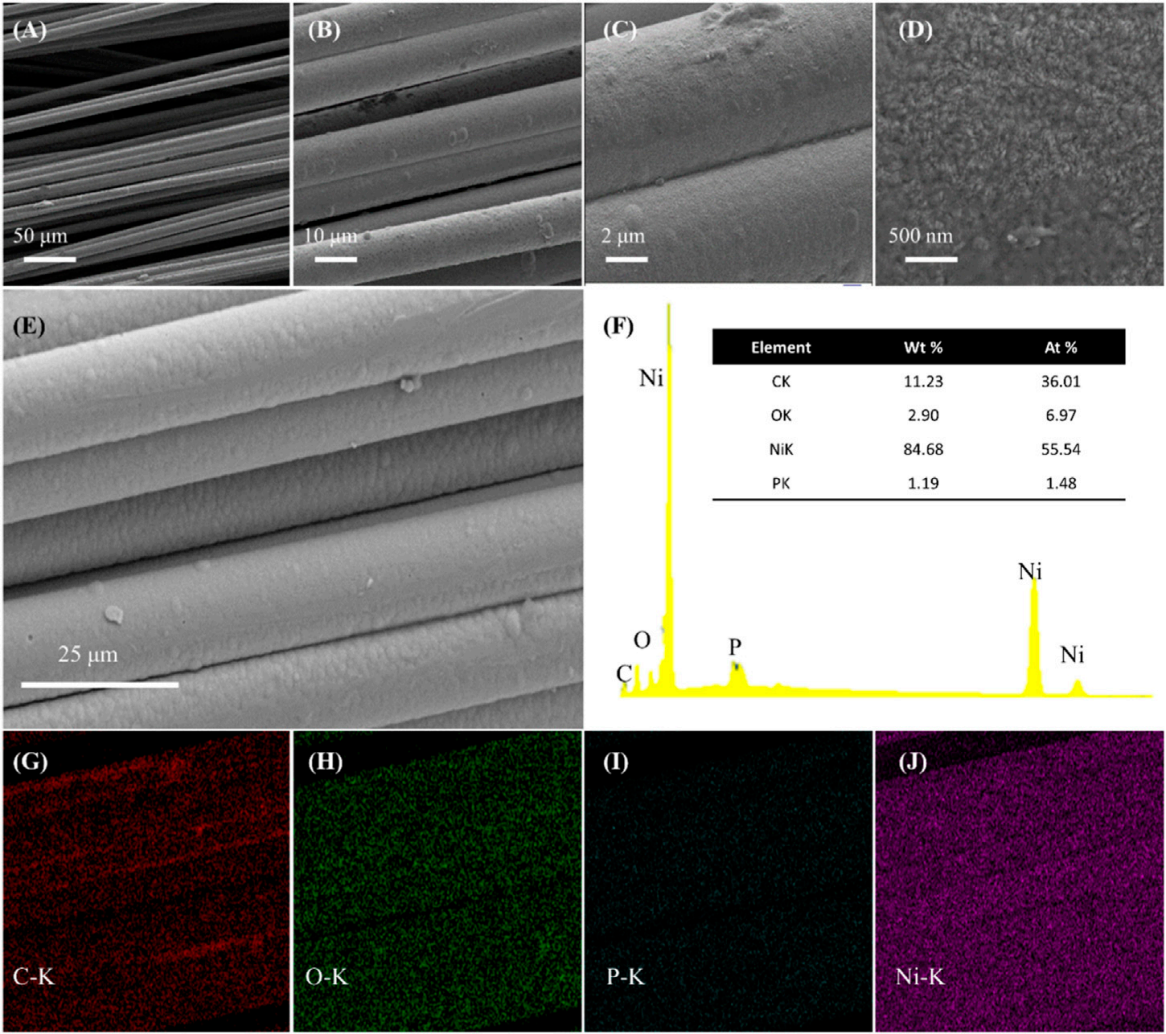
**(A–E)** SEM images of Ni-P/CF at different amplification. **(F–J)** The EDX spectrum and its corresponding elemental mapping of carbon, oxygen, phosphorus, and nickel elements.

Supplementary Figure S1A, B shows the macroscopic morphology of CF and Ni-P/CF. As shown in Supplementary Figure S1B, the surface of Ni-P/CF presents obvious metallic luster, which intuitively reflects the success of electroless plating. SEM images at different magnifications are shown in Figures 1A–E. It can be seen that Ni-P/CF is cylindrical with a diameter of about 10 μm. There is a uniform and compact coating on the surface without obvious cracks, which is beneficial to increase the electric conductivity of carbon fiber. The energy dispersion X-ray (EDX) analysis shows the elemental composition (Figures 1F–J). The content of Ni is the main part (atomic ratio of 55.54%), relative to P (atomic ratio of 1.48%), C (atomic ratio of 36.01%), and O (atomic ratio of 6.97%). In addition, each element is well distributed on the surface of the composite material, indicating that the catalyst material is uniformly deposited.

**FIGURE 2:**
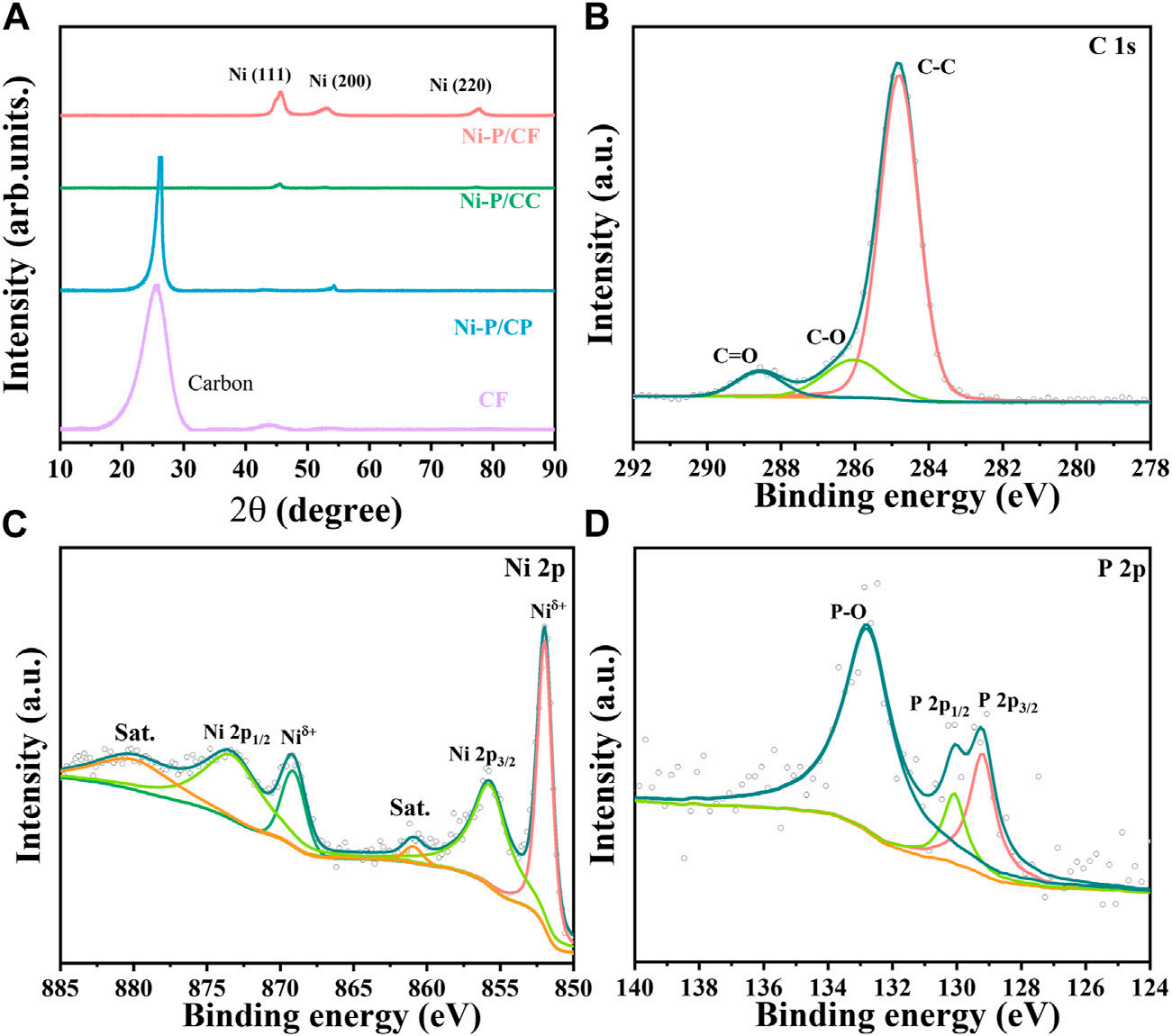
**(A)** XRD patterns of Ni-P/CF, Ni-P/CC, Ni-P/CP, and CF. High-resolution XPS spectra of **(B)** C 1s, **(C)** Ni 2p, and **(D)** P 2p of Ni-P/CF.

To further study the structure of the prepared materials, XRD analysis was adopted (Figure 2A), which further proved the successful growth of Ni-P materials on carbon fibers. A distinct peak at 25.5° can be observed on pristine CF, which is characteristic of carbon. After electroless nickel plating, the catalyst produces characteristic peaks at 45.5°, 52.9°, and 77.8°, which can be attributed to the Ni(111), Ni(200), and Ni(220), respectively. Compared with Ni-P/CC and Ni-P/CP, the peak intensity of Ni-P/CF is the highest and broadest, reflecting the most successful plating of the Ni-P on the CF surface. However, no diffraction peaks related to Ni-P were found, which may be because the Ni-P deposited on the carbon fiber is amorphous. The doping of P changes the crystal structure of nickel, making the coating an amorphous structure (Liu et al., 2020). The high-resolution spectra of elements were tested and analyzed. As exhibited in Supplementary Figure S2, the XPS survey spectrum of Ni-P/CF demonstrates the existence of C, O, Ni, and P elements, which is consistent with EDS analysis. Based on C 1s (284.8 eV), the data obtained were fitted and corrected. The C1s spectrum of Ni-P/CF (Figure 2B) shows three peaks at 284.8, 286, and 288.6 eV, which can be deconvolution into C-C, C-O, and C=O, respectively. In Figure 2C, the Ni 2p spectrum of Ni-P/CF is fitted into six peaks, among which the peak centered at 880 and 860.9 eV are the satellite peaks. The binding energies of 869.1 and 852 eV correspond to Ni in the metallic state, and the other two peaks at 873.3 and 855.7 eV are attributed to bivalent Ni, corresponding to Ni 2p_1/2_ and Ni 2p_3/2_, respectively. Their existence is the result of the oxidation of the material surface caused by not being placed in a vacuum condition (Bhanja et al., 2021). As shown in Figure 2D, P 2p has three characteristic peaks at 132.8, 130.1, and 129.2 eV. The former is also attributed to the surface oxidation of the material, and the latter two peaks are respectively assigned to P 2p_1/2_ and P 2p_3/2_, indicating the formation of a phosphate-metal bond.

### 3.2 Electrocatalytic performance for UOR

**FIGURE 3:**
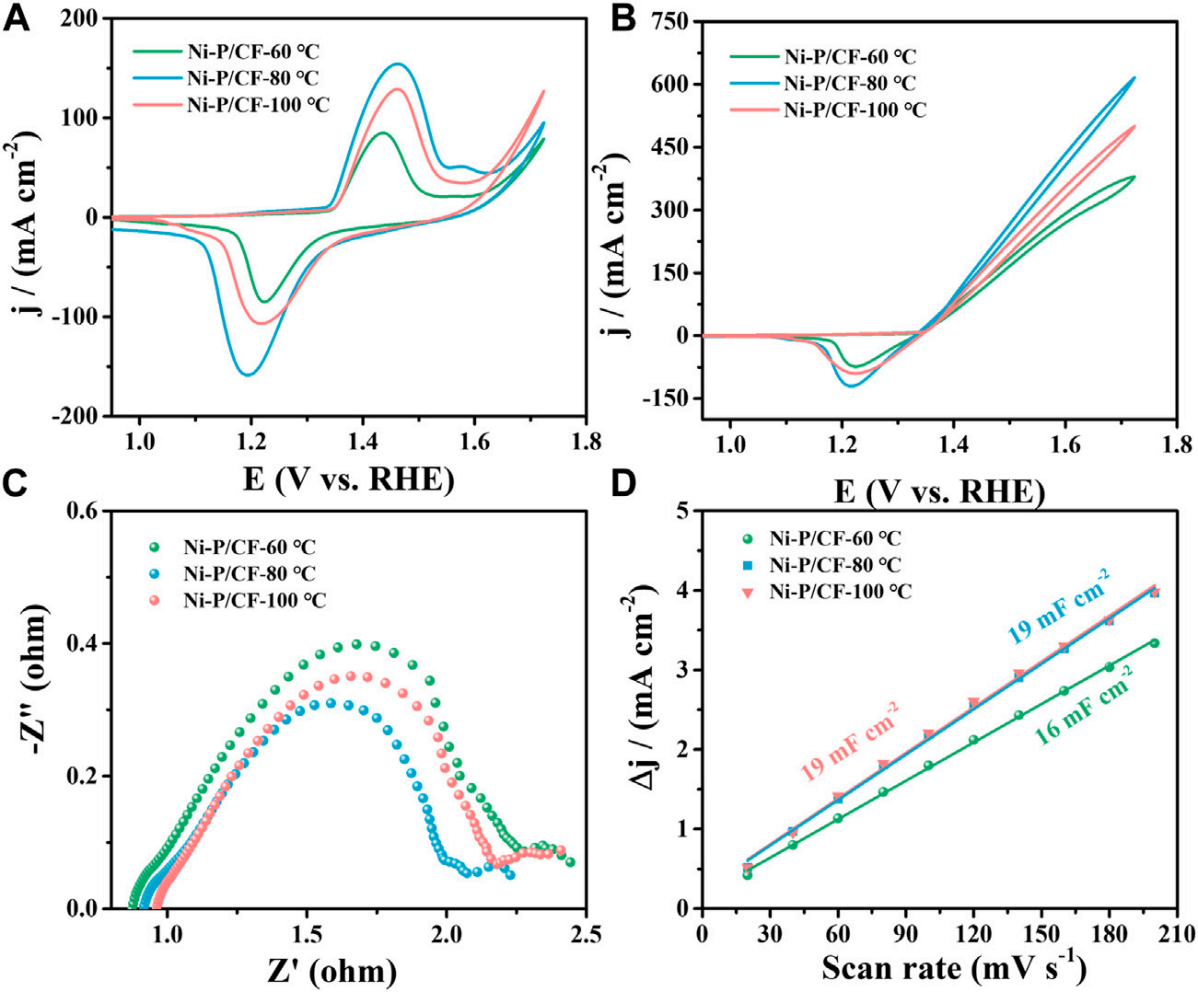
**(A)** CVs of Ni-P/CF-60–100 in 1 M KOH. **(B)** CVs, **(C)** EIS, and **(D)** C_dl_ value of Ni-P/CF-60–100 in 1 M KOH containing 0.33 M urea.

In this work, the effects of heat treatment temperature, Ni/P molar ratio, and pH value of plating solution on UOR activity were firstly investigated to determine the optimal process conditions for electroless nickel plating on carbon fibers. The specific process parameters were statistically shown in Supplementary Table S1. Firstly, the heat treatment temperature in the electroless plating process was explored. Figures 3A, B show the CVs of the catalysts in the presence and absence of urea. It is obvious that the introduction of urea in the electrolyte greatly increased the current density. The maximum current density of Ni-P/CF-80 is 616.60 mA cm^-2^, which is significantly bigger than that of Ni-P/CF-60 (380 mA cm^-2^) and Ni-P/CF-100 (500.20 mA cm^-2^). Electrochemical impedance spectroscopy (EIS) was used to further explore the kinetic performance of the catalysts (Figure 3C). The diameter of the semicircle can represent the charge transfer resistance (R_ct_), which is used to evaluate the electrochemical reaction rate (Wu et al., 2014; Galal et al., 2020; Hatami et al., 2021). The R_ct_ value of Ni-P/CF-80 is 1.38 Ω, which is smaller than that of Ni-P/CF-60 (2.27 Ω), and Ni-P/CF-100 (1.95 Ω), suggesting its faster charge transfer capability. In addition, to evaluate the electrochemical surface area (ECSA) of the samples, the specific double-layer capacitor (C_dl_) based on the CV was studied in the non-Faraday region at a scanning rate ranging from 20 to 200 mV s^-1^ (Supplementary Figures S3A–C). The C_dl_ value of Ni-P/CF-80 is 19 mF cm^-2^, which is comparable to the contrast catalysts of Ni-P/CF-60 (16 mF cm^-2^) and Ni-P/CF-100 (19 mF cm^-2^), indicating that they have sufficient active sites (Figure 3D). The i-t curves of the electrode materials at different potentials were also tested to compare their catalytic stability (Supplementary Figures S3D–F). For instance, ignoring the effect of the initial reaction kinetics, the calculated inactivation rates (Mohamed and Liu, 2019) (j_100s_ -j_1200s_)/j_100s_) of Ni-P/CF-80 is 5.5%, which is smaller than Ni-P/CF-60 (21.4%) and Ni-P/CF-100 (18.8%), indicating Ni-P/CF has more excellent stability than the other materials at 1.52 V (vs. RHE). After 1,200 s of the i-t test, the current density of Ni-P/CF-80 is 185.78 mA cm^-2^, which is bigger than Ni-P/CF-60 (114.68 mA cm^-2^) and Ni-P/CF-100 (127.6 mA cm^-2^), which is further suggesting Ni-P/CF has also outstanding activity while maintaining stability.

**FIGURE 4:**
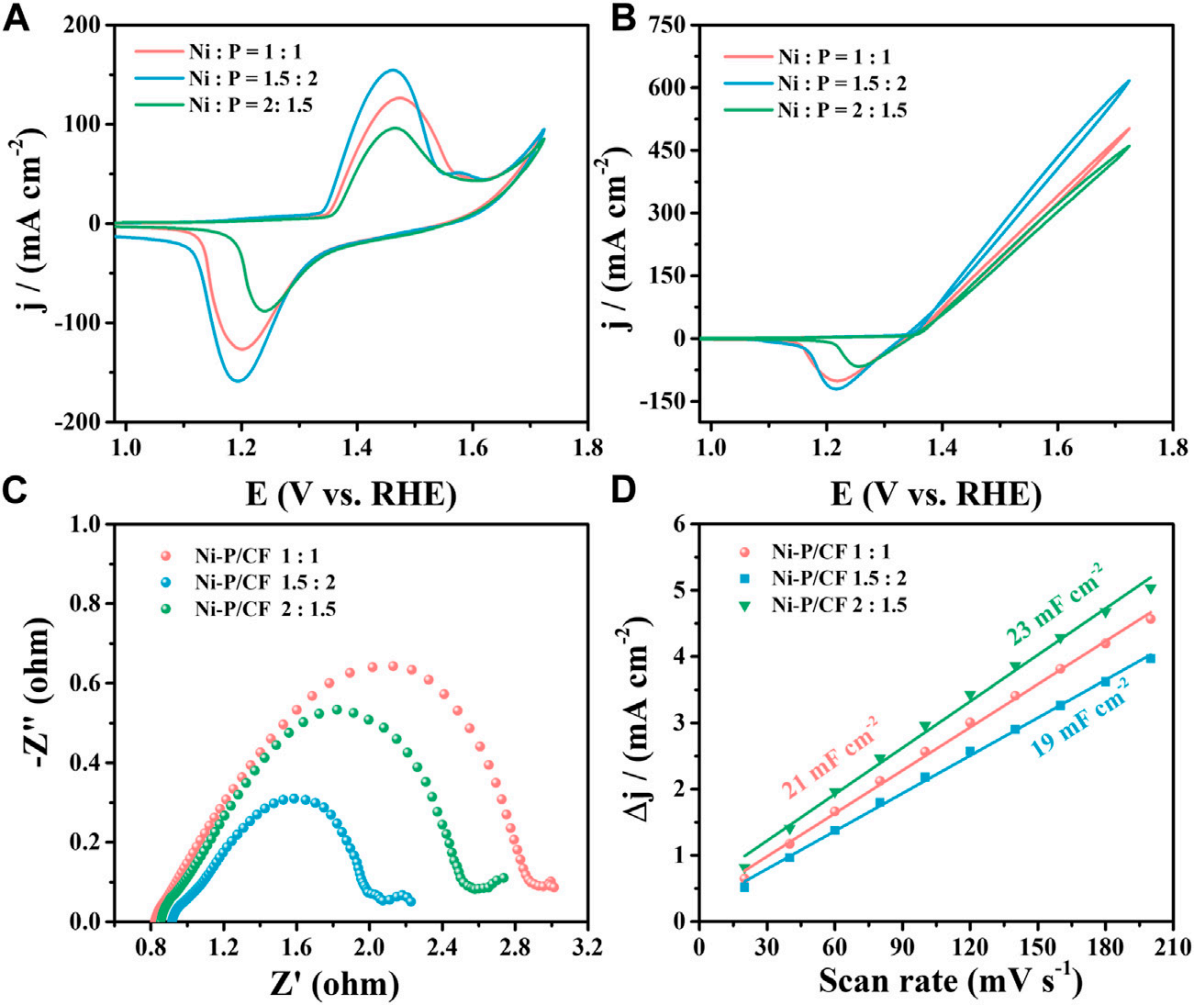
**(A)** CVs of Ni-P/CF with different Ni/P molar ratios in 1 M KOH. **(B)** CVs, **(C)** EIS, and **(D)** C_dl_ value of Ni-P/CF with different Ni/P molar ratios in 1 M KOH containing 0.33 M urea.

Whereafter, we also investigated the effect of the Ni/P ratio of the electroless plating solution on the UOR activity (Figure 4). Varying Ni/P molar ratio, corresponding to Ni-P/CF-80 1.5:2, Ni-P/CF-80 1:1 and Ni-P/CF-80 2:1.5 were obtained and compared. When the ratio of Ni/P is 1.5:2, Ni-P/CF displays superior UOR activity with the maximum current density of 616.60 mA cm^-2^, smaller R_ct_ value of 1.38 Ω and a bigger C_dl_ value of 19 mF cm^-2^ in 1 M KOH containing 0.33 M urea. When the ratio of Ni/P is 1:1 (Ni/P = 2:1.5), Ni-P/CF displays moderate UOR activity with the maximum current density of 502.20 mA cm^-2^(Ni/P = 2:1.5, 460.40 mA cm^-2^), smaller R_ct_ value of 4.84 Ω (Ni/P = 2:1.5, 11.42 Ω) and bigger C_dl_ value of 21 mF cm^-2^ (Ni/P = 2:1.5, 23 mF cm^-2^). In addition, the inactivation rates of Ni-P/CF 1:1 and Ni-P/CF 2:1.5 are 12.9% and 11.9% (Supplementary Figure S5).

**FIGURE 5:**
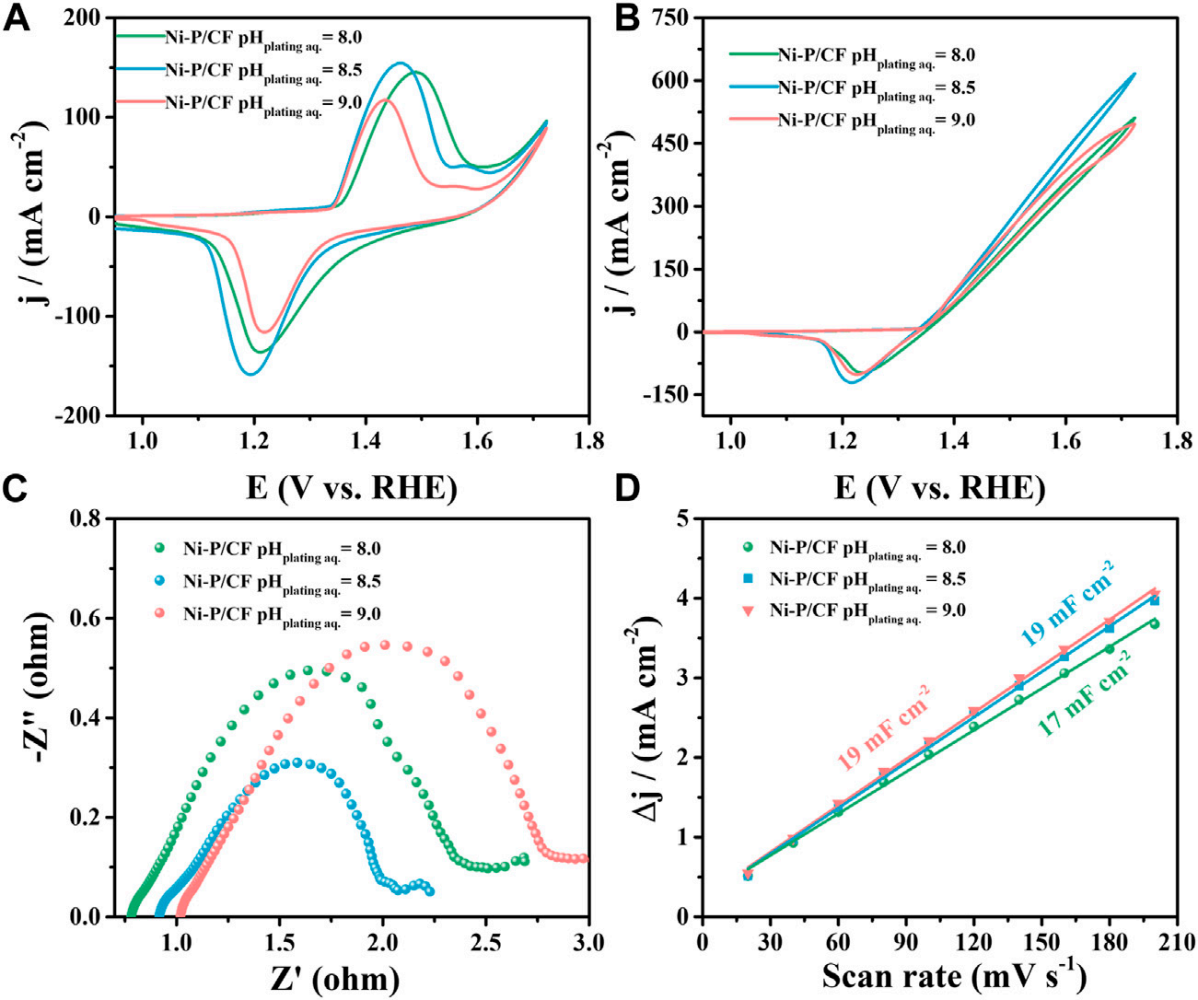
**(A)** CVs of Ni-P/CF pHplating aq. = 8–9 in 1 M KOH. **(B)** CVs, **(C)** EIS, and **(D)** Cdl value of Ni-P/CF pHplating aq. = 8–9 in 1 M KOH containing 0.33 M urea.

Finally, we further explored the effect of the pH of the electroless plating solution on the UOR activity (Figure 5). Varying pH of the electroless plating solution, corresponding to Ni-P/CF pH _plating aq._ = 8, Ni-P/CF pH _plating aq._ = 8.5 and Ni-P/CF pH _plating aq._ = 9 were prepared and compared. When the pH value of the electroless plating solution is 8.5, Ni-P/CF displays superior UOR activity with the maximum current density of 616.60 mA cm^-2^, smaller R_ct_ value of 1.38 Ω, and a bigger C_dl_ value of 19 mF cm^-2^ in 1 M KOH containing 0.33 M urea. When the pH value of the electroless plating solution is 8 (pH = 9), Ni-P/CF exhibit moderate UOR activity with the maximum current density of 510.80 mA cm^-2^ (pH _plating aq._ = 9, 496.20 mA cm^-2^), smaller R_ct_ value of 3.89 Ω (pH _plating aq._ = 9, 5.39 Ω) and bigger C_dl_ value of 17 mF cm^-2^ (pH _plating aq._ = 9, 19 mF cm^-2^). As exhibited in Supplementary Figure S5, the i-t curves of Ni-P/CF pH _plating aq._ = 8 and Ni-P/CF pH _plating aq._ = 9 exhibited that their inactivation rates are 14.8% and 8.2%, respectively (1.52 V vs. RHE). To sum up, the experimental result display that the optimal treatment temperature of the plating solution is 80°C, the optimal Ni/P ratio is 1.5:2, and the optimal pH value is 8.5.

**FIGURE 6:**
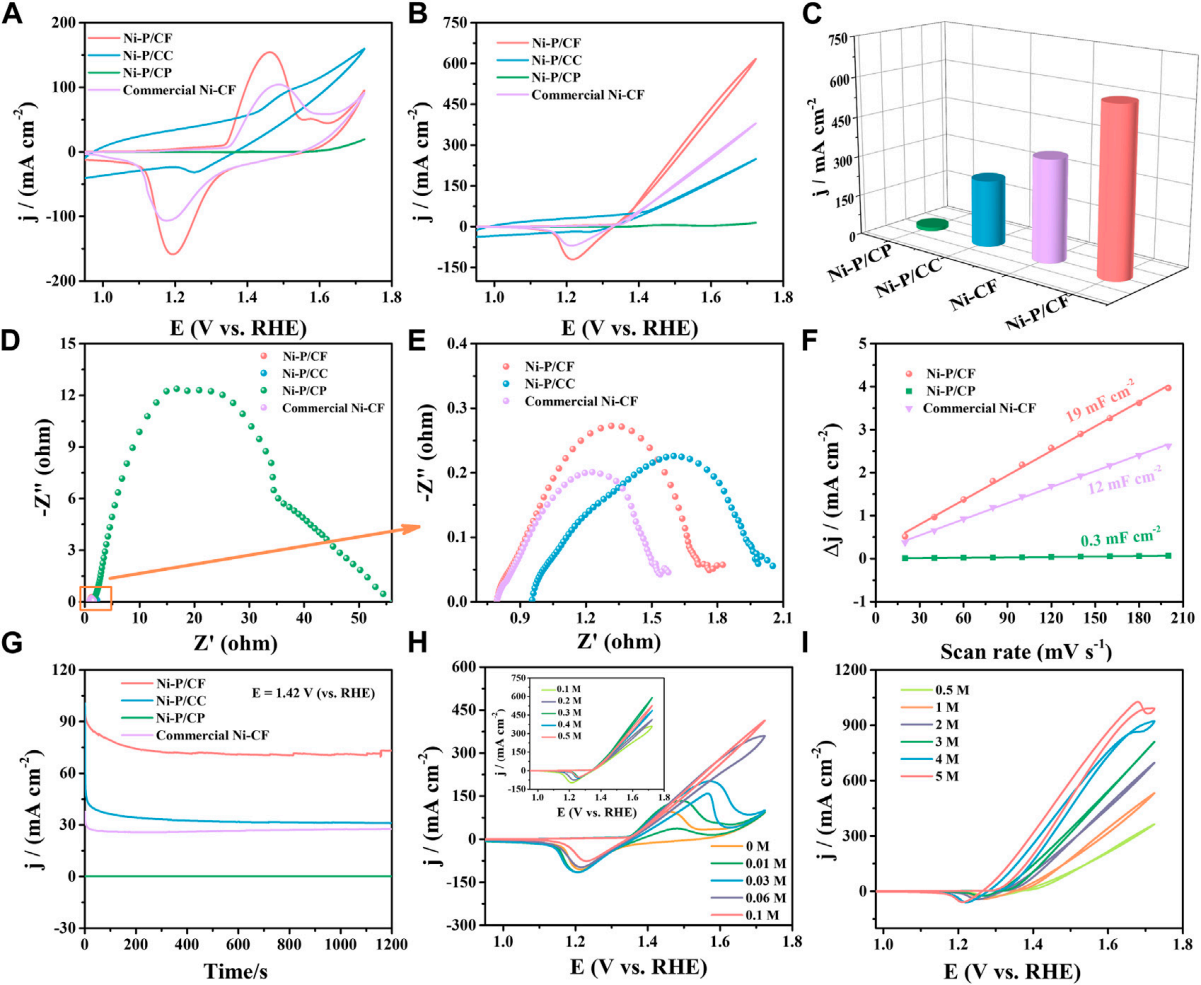
**(A)** CVs of samples in 1 M KOH. **(B)** CVs of samples in 1 M KOH with 0.33 M urea. **(C)** Comparison of potential at the maximum current density among the catalysts. **(D–E)** EIS, **(F)** C_dl_ linear fitting and calculation, and **(G)** i-t curves of different materials in 1 M KOH with 0.33 M urea. **(H)** CVs of the Ni-P/CF at urea content from 0 M to 0.1 M (inset: CVs of the Ni-P/CF at urea content from 0.1 M to 0.5 M). **(I)** CVs of the Ni-P/CF at KOH concentrations from 0.5 M to 5 M in 0.33 M urea.

Hence, the UOR property of Ni-P/CF and contrast catalysts with the optimal preparation conditions of treatment temperature of 80°C, the Ni/P ratio of 1.5:2, and the pH value of 8.5 was further investigated. Figures 6A, B reveals the CV curves for UOR of Ni-P/CF and contrast catalysts in 1.0 M KOH solution with the presence/absence of 0.33 M urea. As exhibited in Figure 6B, the Ni-P/CF displays a prominently increased current density of 616.60 mA cm^-2^ at a potential of 1.72 V (vs. RHE), which is about 1.62, 43.01, and 2.48 times higher than that of commercial Ni-CF (380.00 mA cm^-2^), Ni-P/CP (14.33 mA cm^-2^) and Ni-P/CC (248.60 mA cm^-2^) (Figure 6C). Specifically, compared with the CVs of Ni-P/CF in 1.0 M KOH, the current density increased dramatically in 1.0 M KOH with 0.33 M urea, suggesting a superior UOR activity (Supplementary Figure S7). Ni-P/CF displays superior UOR performance with a potential of 1.40 V to obtain a current density of 100 mA cm^-2^. The commercial Ni-CF shows a moderate UOR performance with a potential of 1.45 V to reach a current density of 100 mA cm^-2^. The Ni-P/CC exhibit poor UOR activity with a potential of 1.50 V to acquire a current density of 100 mA cm^-2^. EIS was employed to further comprehend the dynamics for UOR (Figure 6D). EIS results exhibit that the R_ct_ value of Ni-P/CP (35.06 Ω) is much greater than that of the other three materials, indicating inferior electrical conductivity and an inefficient charge transfer during UOR. Enlarge the EIS spectra of Ni-P/CC, commercial Ni-CF, and Ni-P/CF show small R_ct_ values (2.71 Ω, 0.79 Ω, and 1.38 Ω respectively), indicating they have effective electron transfer for UOR (Figure 6E). The C_dl_ values was measurement to estimate the electrochemical surface area (ECSA) (Figure 6F). The C_dl_ values of Ni-P/CF is 19 mF cm^-2^, which is bigger than that of Ni-P/CP (0.3 mF cm^-2^), commercial Ni-CF (12 mF cm^-2^), suggesting that large exposed active species of Ni-P/CF.

In practical applications, the electrochemical stability of catalysts in alkaline media is also an important criterion. i-t curves were executed to explore the stability for Ni-P/CF and the contrast material at the potential of 1.42 V vs. RHE (Figure 6G). As exhibited in Figure 6G, the current density of Ni-P/CF, Ni-P/CC, and commercial Ni-P/CF have a substantial decay in the initial stage, which is because of the transformation of Ni(OH)_2_ to intermediate NiOOH during the sustaining urea electrolytic. After that, the kinetics of the intermediate removal of the reactants and the urea oxidation reaction remain consistent, and the current density keeps a small change (Yang et al., 2021). The current density of Ni-P/CF is significantly higher than that of other materials, which reflects the faster interfacial electron transfer of Ni-P/CF. Supplementary Figure S9 shows the i-t curves of different electrodes at different potentials (1.47 V, 1.52 V vs. RHE). Figure 6H displays the CVs of Ni-P/CF in 1 M KOH with different urea. Then, we investigated the effect of urea concentration on the kinetics of Ni-P/CF for urea oxidation in 1 M KOH containing urea (0.–0.1 M, 0.1–0.5 M) (Figure 6H). As shown in Figure 6H, with the increase of urea content from 0 to 0.1 M, the position of the oxidation peak of the catalyst gradually shifts to the right, and the maximum current density gradually increases because the NiO(OH) active species start to catalytic oxidation of urea (Singh and Schechter, 2017; Alex et al., 2021). This result indicates that there is a diffusion controlled on the surface of Ni-P/CF with urea content from 0 m to 0.1 M (Vedh et al., 2012). In the inset of Figure 6H, the current density was significative increased with the increase of urea content to 0.33 M, and then the current density was decreased when the content of urea was increased to 0.5 M due to the equilibration adsorption of urea on the surface of Ni-P/CF. This result may be due to the lack of OH^−^ ions in the catalyst for the oxidation of Ni^2+^ to Ni^3+^ on the electrode surface. It can also be proved by the trend that the current density increases with the increase of KOH concentration in Figure 6I. In addition, urea hydrolysis is also a major factor, which consumes urea while generating gas CO_2_ and N_2_, whose diffusion to the surface of electrode hinder the electrode respond (Song et al., 2017).

**FIGURE 7:**
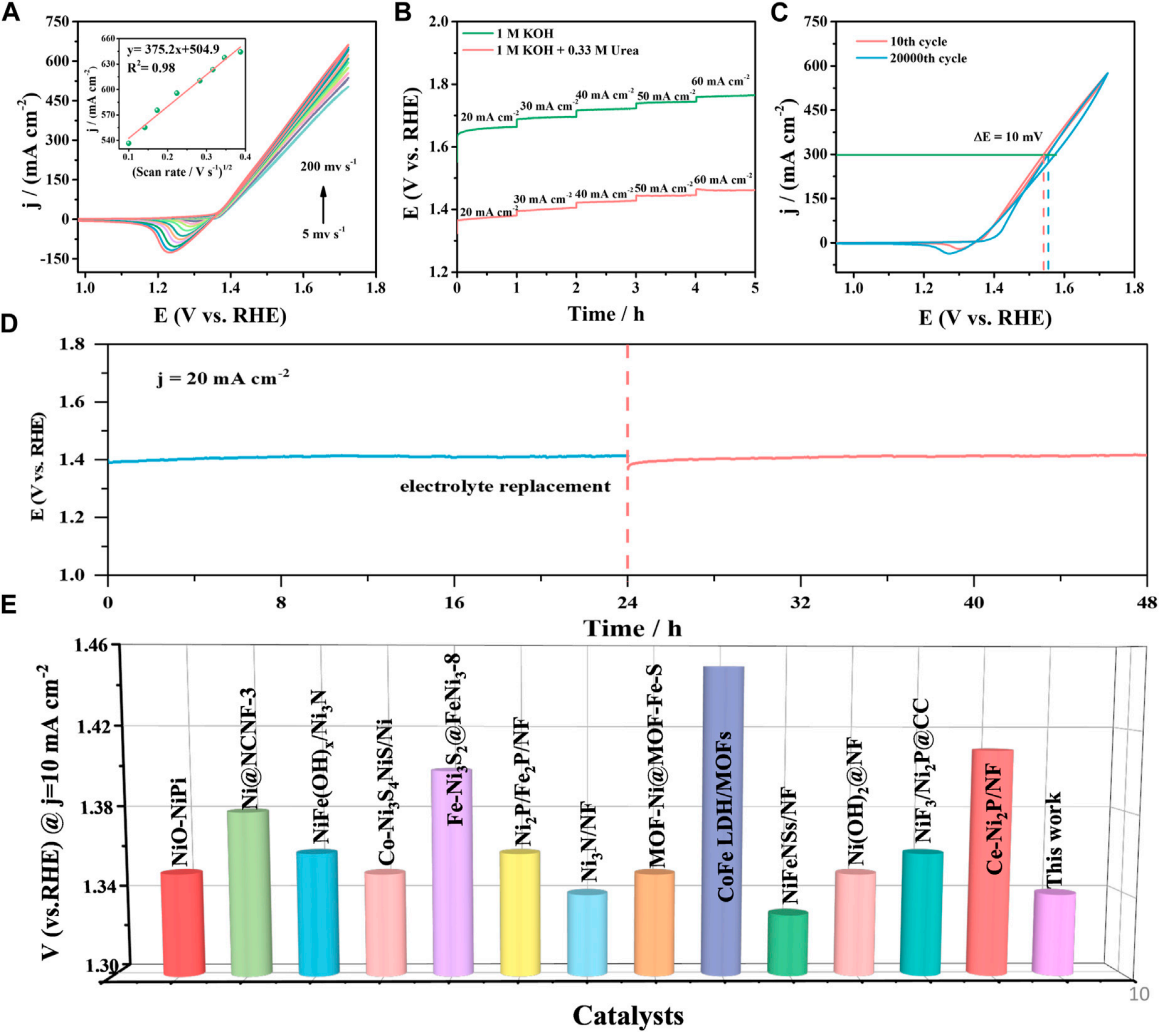
**(A)** CVs of Ni-P/CF at different scanning rates. **(B)** Multi-current curve of Ni-P/CF in 1 M KOH with and without 0.33 M urea. **(C)** CVs recorded at the 10th cycle and 20000th cycle. **(D)** The V-T curve of Ni-P/CF in 1 M KOH containing 0.33 M urea. **(E)** Comparison of the potentials at j = 10 mA cm^-2^ among the reported UOR catalyst.

Figure 7A exhibits the CV curve of Ni-P/CF in 1 M KOH containing 0.33 M urea at different scanning rates (5–200 mV s^-1^). The current density of the Ni-P/CF increased with the increase in scan rate. For instance, the maximum current density of Ni-P/CF was increased from 503.60–662.20 mA cm^-2^ with the scan rate increasing from 5 to 200 mV s^-1^. This result indicates that the irreversible reaction on the surface of Ni-P/CF was faster (Bai et al., 2020). Furthermore, there is a linear characteristic between the square root of the scan rate and the current density (Figure 7A, inset), which further imply that the electron transport mechanism of the catalytic process is regulated by the diffusion process. Figure 7B displays the multi-current curves of Ni-P/CF. The relevant potentials are recorded with an increment of 10 mA cm^-2^ with a current density range from 20 to 60 mA cm^-2^. For example, the current density executes at 20 mA cm^-2^ for 3,600 s in the first stage. Compared with the multi-current curves of Ni-P/CF in 1 M KOH, Ni-P/CF exhibits a very low potential in 1 M KOH with 0.33 M urea, implying good UOR activity. Besides, the potential promptly levels off and executes gradually stable for the remaining time. The long-term stability is a crucial criterion to estimate the UOR electrocatalyst for practical applications. The long-term stability is a crucial criterion to estimate the UOR electrocatalyst for practical applications (Figure 7C). The stability of the Ni-P/CF was tested by continuous potential cycling in 1 M KOH solution containing 0.33 M urea. Between 10th and 20000th cycles, the CV curves nearly remain unchanged, which proves the outstanding durability of the Ni-P/CF. Amazedly, after 20000 cycles, the potential only declined 10 mV compared with the 10th curve, demonstrating its excellent electrochemical reproducibility, and cycling stability. The result can also be further verified by executing a continuous chronoamperometric.

As displayed in Figure 7D, the potential of the Ni-P/CF electrode was still maintained almost constant in 48 h period at a constant current density of 20 mA cm^-2^, indicating the splendid durability of the Ni-P/CF electrode in an alkaline environment for the UOR. The applied voltage showed a slight rise at 24 h, which may be due to the consumption of the active substance NiOOH or the poisoning of the catalyst caused by CO_2_ generated by the complete oxidation of urea at the anode (Sayed et al., 2019; Xu et al., 2021). Encouragingly, the catalyst almost recovered its original activity by changing the electrolyte. The UOR potential of Ni-P/CF can also compete with many excellent related catalysts. The specific properties of these related catalysts are listed in Supplementary Table S2 and reflected in Figure 7E, which intuitively demonstrates the excellent UOR performance of Ni-P/CF.

**FIGURE 8:**
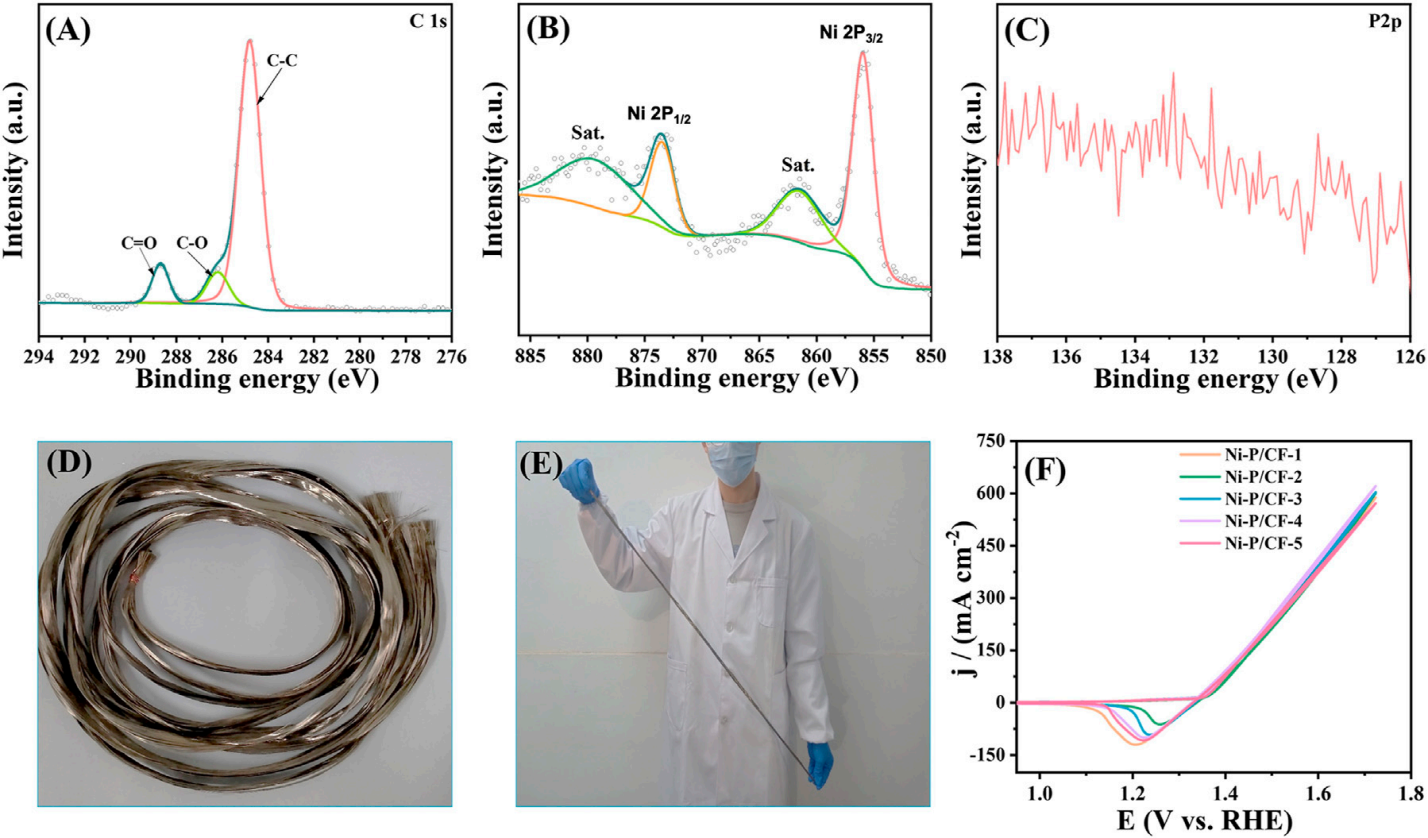
High-resolution XPS spectra of **(A)** C 1s, **(B)** Ni 2p, **(C)** P 2p of Ni-P/CF after 20000 cycles CVs test. **(D–E)** Illustration of 100 cm Ni-P/CF. **(F)** CV sampling test of 100 cm Ni-P/CF.

The XPS was employed to further explore the chemical states for Ni-P/CF after 20000 cycles CVs test (Figures 8A–C). As exhibited in the spectra of Ni 2p, the peak centered at 879.35 and 861.48 eV can be ascribed to the satellite peaks, and the peaks at 873.47 and 855.93 eV are ascribed to Ni 2p_1/2_ and Ni 2p_3/2_, respectively, which correspond to Ni-OH bonds (Liu et al., 2021) In the spectra of P 2p, no characteristic peaks appear, indicating that P was dissolved during the test in the electrolyte. In addition, to explore the practical application potential of the scheme, we successfully synthesized 100 cm nickel-coated carbon fiber (Figures 8D, E). Then, we cut randomly five pieces of carbon fiber to measure the reproducibility for UOR activity (Figure 8F). The test results show that the Ni-P/CF has good reproducibility and potential for mass production. The realization of a scale-up laboratory provides an important experimental basis and success confidence for the leap from laboratory scale to industrial production.

**FIGURE 9:**
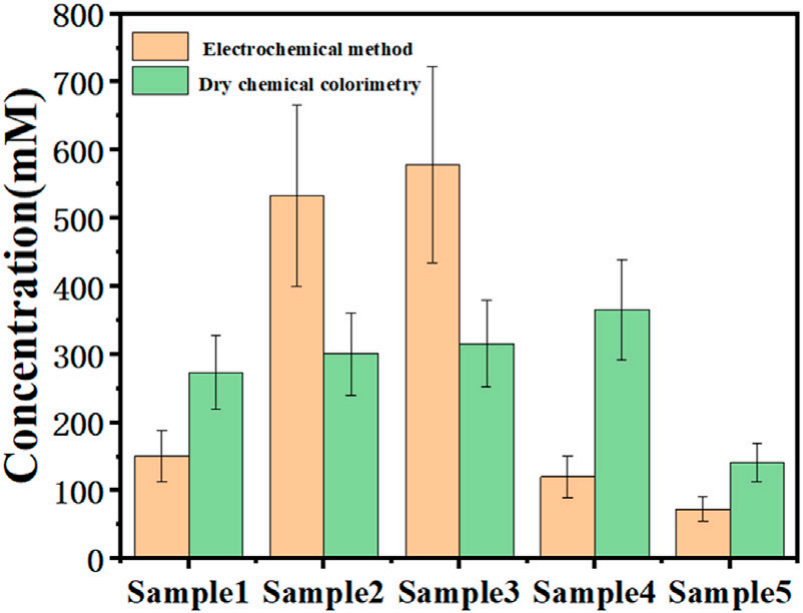
Urea detection in real urine with different methods.

The synthetic electrode was also tested for urea content in real urine. Urine was diluted 10,000 times and electrochemical testing was performed. The results of electrochemical tests and dry chemical colorimetry used in conventional medicine were compared. In order to ensure uniform test conditions, no preservatives were added to the dry chemical colorimetric test, as can be seen in Figure 9. The accuracy of dry chemical analysis is limited by a variety of interference factors, including the concentration of drug or nutrient metabolites in patient urine samples, changes in the concentration of certain metabolites caused by long-term exposure to light or room temperature environment, *etc.* Among them, the interference of vitamin C is the most obvious. However, the oxidation peak of vitamin C in electrochemical detection is very different from that of urea, so the influence of vitamin C can be avoided in electrochemical detection, and the results are more accurate. It can be seen from the test results in the manuscript that the electrochemical detection in this study basically had the same changing trend as the medical detection, and had high credibility. At the specific application level, the detection and data summary of a large number of actual samples are also required. The detection of urea with the electro-probe in practical application needs more correction. This study provides preliminary basic research for practical applications.

## 4 Conclusion

In conclusion, Ni-P/CF has been successfully grown directly on CF surface by a sample hydrothermal technique. The UOR property measurement exhibits that the optimal treatment temperature of the plating solution is 80°C, the optimal Ni/P ratio is 1.5:2, and the optimal pH value is 8.5. The as-synthesized Ni-P/CF shows superior UOR performance with a maximum current density of 616.60 mA cm^-2^ in 1.0 M KOH with 0.33 M urea. Surprisingly, the cycle stability test of Ni-P/CF displays superior stability with the △V of 10 mV at the current density of 300 mA cm^-2^ between 10th and 20000th cycles. Furtherly, the robust durability was also demonstrated by 48 h V-T test at 20 mA cm^-2^. Importantly, large-scale synthesis of Ni-P/CF can be achieved according to the method in this work. As a demonstration, we successfully synthesized Ni-P/CF with a length of 100 cm. This large-scale catalyst synthesis undoubtedly makes practical industrial and medical production possible and provides an effective strategy.

## Data Availability

The original contributions presented in the study are included in the article/Supplementary Material, further inquiries can be directed to the corresponding author.

## References

[B1] Alex C. Shukla G. John N. S. (2021). Introduction of surface defects in NiO with effective removal of adsorbed catalyst poisons for improved electrochemical urea oxidation. Electrochim. Acta 385, 138425. 10.1016/j.electacta.2021.138425

[B2] Amstutz V. Katsaounis A. Kapalka A. Comninellis C. Udert K. M. (2012). Effects of carbonate on the electrolytic removal of ammonia and urea from urine with thermally prepared IrO_2_ electrodes. J. Appl. Electrochem. 42 (9), 787–795. 10.1007/s10800-012-0444-y

[B3] Bai J. Jia N. Jin P. Chen P. Jiang J.-X. Zeng J.-H. (2020). Metal-organic interface engineering for boosting the electroactivity of Pt nanodendrites for hydrogen production. J. energy Chem. 51, 105–112. 10.1016/j.jechem.2020.03.054

[B4] Bhanja P. Kim Y. Paul B. Kaneti Y. V. Alothman A. A. Bhaumik A. (2021). Microporous nickel phosphonate derived heteroatom doped nickel oxide and nickel phosphide: efficient electrocatalysts for oxygen evolution reaction. Chem. Eng. J. 405, 126803. 10.1016/j.cej.2020.126803

[B5] Camposeco R. Hinojosa-Reyes M. Zanella R. (2021). Highly efficient photocatalytic hydrogen evolution by using Rh as co-catalyst in the Cu/TiO_2_ system. Int. J. Hydrogen Energy 46 (51), 26074–26086. 10.1016/j.ijhydene.2021.01.216

[B6] Choi B.-K. Park S.-J. Seo M.-K. (2020). Effect of processing parameters on the thermal and electrical properties of electroless nickel-phosphorus plated carbon fiber heating elements. C 6 (1), 6. 10.3390/c6010006

[B7] Fayyad E. M. Abdullah A. M. Hassan M. K. Mohamed A. M. Jarjoura G. Farhat Z. (2018). Recent advances in electroless-plated Ni-P and its composites for erosion and corrosion applications: a review. Emergent Mater 1 (1-2), 3–24. 10.1007/s42247-018-0010-4

[B8] Feng Y. Wang X. Huang J. Dong P. Ji J. Li J. (2020). Decorating CoNi layered double hydroxides nanosheet arrays with fullerene quantum dot anchored on Ni foam for efficient electrocatalytic water splitting and urea electrolysis. Chem. Eng. J. 390, 124525. 10.1016/j.cej.2020.124525

[B9] Galal A. Atta N. F. Hefnawy M. A. (2020). Voltammetry study of electrocatalytic activity of lanthanum nickel perovskite nanoclusters-based composite catalyst for effective oxidation of urea in alkaline medium. Synth. Mater. 266, 116372. 10.1016/j.synthmet.2020.116372

[B10] Gao H. Zhang B. Qiu C. Xiao Y. Wang W. (2021). Enhanced interfacial interactions between Ni2Fe (hydroxy)oxides and oxygen-modified carbon substrate for efficient oxygen evolution reaction. Ionics 27 (9), 3987–3994. 10.1007/s11581-021-04174-y

[B11] Guo F. Cao D. Du M. Ye K. Wang G. Zhang W. (2016). Enhancement of direct urea-hydrogen peroxide fuel cell performance by three-dimensional porous nickel-cobalt anode. J. Power Sources 307, 697–704. 10.1016/j.jpowsour.2016.01.042

[B12] Hatami E. Toghraei A. Barati Darband G. (2021). Electrodeposition of Ni-Fe micro/nano urchin-like structure as an efficient electrocatalyst for overall water splitting. Int. J. Hydrogen Energy 46 (14), 9394–9405. 10.1016/j.ijhydene.2020.12.110

[B13] Hu S. Wang S. Feng C. Wu H. Zhang J. Mei H. (2020). Novel MOF-derived nickel nitride as high-performance bifunctional electrocatalysts for hydrogen evolution and urea oxidation. ACS Sustain. Chem. Eng. 8 (19), 7414–7422. 10.1021/acssuschemeng.0c01450

[B14] King R. L. Botte G. G. (2011). Investigation of multi-metal catalysts for stable hydrogen production via urea electrolysis. J. Power Sources 196 (22), 9579–9584. 10.1016/j.jpowsour.2011.06.079

[B15] Krajewska B. (2009). Ureases I. Functional, catalytic and kinetic properties: a review. J. Mol. Catal. B Enzym. 59 (1-3), 9–21. 10.1016/j.molcatb.2009.01.003

[B16] Li J. Li J. Liu T. Chen L. Li Y. Wang H. (2021a). Deciphering and suppressing over-oxidized nitrogen in nickel-catalyzed urea electrolysis. Angew. Chem. Int. Ed. Engl. 60 (51), 26860–26866. 10.1002/ange.202107886 34553818

[B17] Li Q. Li X. Gu J. Li Y. Tian Z. Pang H. (2020). Porous rod-like Ni_2_P/Ni assemblies for enhanced urea electrooxidation. Nano Res. 14 (5), 1405–1412. 10.1007/s12274-020-3190-1

[B18] Li S. Fan J. Li S. Ma Y. Wu J. Jin H. (2021b). *In situ*-grown Co3O4 nanorods on carbon cloth for efficient electrocatalytic oxidation of urea. J. Nanostructure Chem. 11 (4), 735–749. 10.1007/s40097-021-00441-6

[B19] Liu H. Zhu S. Cui Z. Li Z. Wu S. Liang Y. (2021). Ni_2_P nanoflakes for the high-performing urea oxidation reaction: linking active sites to a UOR mechanism. Nanoscale 13 (3), 1759–1769. 10.1039/d0nr08025j 33432949

[B20] Liu S. Qiao X. Liu W. Shi S. Qu Y. (2019). Mechanism of ultrasonic treatment under nickel salt solution and its effect on electroless nickel plating of carbon fibers. Ultrason. Sonochem. 52, 493–504. 10.1016/j.ultsonch.2018.12.030 30594522

[B21] Liu X. Zang J. Zhou S. Tian P. Gao H. Song S. (2020b). Electroless deposition of Ni-Cu-P on a self-supporting graphene with enhanced hydrogen evolution reaction activity. Int. J. Hydrogen Energy 45 (27), 13985–13993. 10.1016/j.ijhydene.2020.03.102

[B22] Liu Z. Zhang C. Liu H. Feng L. (2020a). Efficient synergism of NiSe_2_ nanoparticle/NiO nanosheet for energy-relevant water and urea electrocatalysis. Appl. Catal. B-Environ. 276, 119165. 10.1016/j.apcatb.2020.119165

[B23] Lu S. Li H. Tan G. Wen F. Flynn M. T. Zhu X. (2019). Resource recovery microbial fuel cells for urine-containing wastewater treatment without external energy consumption. Chem. Eng. J. 373, 1072–1080. 10.1016/j.cej.2019.05.130

[B24] Mahalik K. Sahu J. N. Patwardhan A. V. Meikap B. C. (2010). Kinetic studies on hydrolysis of urea in a semi-batch reactor at atmospheric pressure for safe use of ammonia in a power plant for flue gas conditioning. J. Hazard. Mat. 175 (1-3), 629–637. 10.1016/j.jhazmat.2009.10.053 19914776

[B25] Mohamed I. M. A. Liu C. (2019). Chemical design of novel electrospun CoNi/Cr nanoparticles encapsulated in C-nanofibers as highly efficient material for urea oxidation in alkaline media. Appl. Surf. Sci. 475, 532–541. 10.1016/j.apsusc.2019.01.003

[B26] Qi Q. Wang Y. Ding X. Wang W. Xu R. Yu D. (2020). High-electromagnetic‐shielding cotton fabric prepared using multiwall carbon nanotubes/nickel-phosphorus electroless plating. Appl. Organomet. Chem. 34 (3), 5434. 10.1002/aoc.5434

[B27] Safeer N M. Alex C. Jana R. Datta A. John N. S. (2022). Remarkable CO_x_ tolerance of Ni^3+^ active species in a Ni_2_O_3_ catalyst for sustained electrochemical urea oxidation. J. Mat. Chem. A 10 (8), 4209–4221. 10.1039/d1ta05753g

[B28] Sayed E. T. Eisa T. Mohamed H. O. Abdelkareem M. A. Allagui A. Alawadhi H. (2019). Direct urea fuel cells: challenges and opportunities. J. Power Sources 417, 159–175. 10.1016/j.jpowsour.2018.12.024

[B29] Sha L. Liu T. Ye K. Zhu K. Yan J. Yin J. (2020b). A heterogeneous interface on NiS@Ni_3_S_2_/NiMoO_4_ heterostructures for efficient urea electrolysis. J. Mat. Chem. A 8 (35), 18055–18063. 10.1039/d0ta04944a

[B30] Sha L. Ye K. Yin J. Zhu K. Cheng K. Yan J. (2020a). *In situ* grown 3D hierarchical MnCo_2_O_4.5_@Ni(OH)_2_ nanosheet arrays on Ni foam for efficient electrocatalytic urea oxidation. Chem. Eng. J. 381, 122603. 10.1016/j.cej.2019.122603

[B31] Singh R. K. Schechter A. (2017). Electroactivity of NiCr catalysts for urea oxidation in alkaline electrolyte. ChemCatChem 9 (17), 3374–3379. 10.1002/cctc.201700451

[B32] Song X. Gao L. Li Y. Chen W. Mao L. Yang J.-H. (2017). Nickel phosphate-based materials with excellent durability for urea electro-oxidation. Electrochim. Acta 251, 284–292. 10.1016/j.electacta.2017.08.117

[B33] Suárez D. Díaz N. Merz K. M. (2003). Ureases: quantum chemical calculations on cluster models. J. Am. Chem. Soc. 125 (50), 15324–15337. 10.1021/ja030145g 14664576

[B34] Sun H.-Y. Xu G.-R. Li F.-M. Hong Q.-L. Jin P.-J. Chen P. (2020). Hydrogen generation from ammonia electrolysis on bifunctional platinum nanocubes electrocatalysts. J. energy Chem. 47, 234–240. 10.1016/j.jechem.2020.01.035

[B35] Sun X. Ding R. (2020). Recent progress with electrocatalysts for urea electrolysis in alkaline media for energy-saving hydrogen production. Catal. Sci. Technol. 10 (6), 1567–1581. 10.1039/c9cy02618e

[B36] Tong Y. Chen P. Zhang M. Zhou T. Zhang L. Chu W. (2018). Oxygen vacancies confined in nickel molybdenum oxide porous nanosheets for promoted electrocatalytic urea oxidation. ACS Catal. 8 (1), 1–7. 10.1021/acscatal.7b03177

[B37] Urbańczyk E. Sowa M. Simka W. (2016). Urea removal from aqueous solutions – a review. J. Appl. Electrochem. 46 (10), 1011–1029. 10.1007/s10800-016-0993-6

[B38] Vedharathinam V. Botte G. G. (2012). Understanding the electro-catalytic oxidation mechanism of urea on nickel electrodes in alkaline medium. Electrochim. Acta 81, 292–300. 10.1016/j.electacta.2012.07.007

[B39] Wang C. Chen L. Li J. Sun S. Ma L. Wu G. (2017). Enhancing the interfacial strength of carbon fiber reinforced epoxy composites by green grafting of poly(oxypropylene) diamines. Compos. Part A Appl. Sci. Manuf. 99, 58–64. 10.1016/j.compositesa.2017.04.003

[B40] Wang S. Zhao L. Li J. Tian X. Wu X. Feng L. (2022). High valence state of Ni and Mo synergism in NiS_2_-MoS_2_ hetero-nanorods catalyst with layered surface structure for urea electrocatalysis. J. energy Chem. 66, 483–492. 10.1016/j.jechem.2021.08.042

[B41] Wang T.-J. Sun H.-Y. Xue Q. Zhong M.-J. Li F.-M. Tian X. (2021). Holey platinum nanotubes for ethanol electrochemical reforming in aqueous solution. Sci. Bull. 66 (20), 2079–2089. 10.1016/j.scib.2021.05.027 36654266

[B42] Wu M.-S. Lin G.-W. Yang R.-S. (2014). Hydrothermal growth of vertically-aligned ordered mesoporous nickel oxide nanosheets on three-dimensional nickel framework for electrocatalytic oxidation of urea in alkaline medium. J. Power Sources 272, 711–718. 10.1016/j.jpowsour.2014.09.009

[B43] Wu T.-H. Hou B.-W. (2021). Superior catalytic activity of α-Ni(OH)_2_ for urea electrolysis. Catal. Sci. Technol. 11 (12), 4294–4300. 10.1039/d1cy00435b

[B44] Xiao C. Li S. Zhang X. MacFarlane D. R. (2017). MnO_2_/MnCo_2_O_4_/Ni heterostructure with quadruple hierarchy: a bifunctional electrode architecture for overall urea oxidation. J. Mat. Chem. A 5 (17), 7825–7832. 10.1039/c7ta00980a

[B45] Xu H. Ye K. Yin J. Zhu K. Yan J. Wang G. (2021a). *In situ* growth of ZIF67 at the edge of nanosheet transformed into yolk-shell CoSe_2_ for high efficiency urea electrolysis. J. Power Sources 491, 229592. 10.1016/j.jpowsour.2021.229592

[B46] Xu X. Guo T. Xia J. Zhao B. Su G. Wang H. (2021b). Modulation of the crystalline/amorphous interface engineering on Ni-P-O-based catalysts for boosting urea electrolysis at large current densities. Chem. Eng. J. 425, 130514. 10.1016/j.cej.2021.130514

[B47] Xue C. Wilson L. D. (2016). Kinetic study on urea uptake with chitosan based sorbent materials. Carbohydr. Polym. 135, 180–186. 10.1016/j.carbpol.2015.08.090 26453866

[B48] Yan W. Wang D. Botte G. G. (2012). Electrochemical decomposition of urea with Ni-based catalysts. Appl. Catal. B-Environ. 127, 221–226. 10.1016/j.apcatb.2012.08.022

[B49] Yang D. Yang L. Zhong L. Yu X. Feng L. (2019b). Urea electro-oxidation efficiently catalyzed by nickel-molybdenum oxide nanorods. Electrochim. Acta 295, 524–531. 10.1016/j.electacta.2018.10.190

[B50] Yang J.-H. Chen M. Xu X. Jiang S. Zhang Y. Wang Y. (2021). CuO-Ni(OH)_2_ nanosheets as effective electro-catalysts for urea oxidation. Appl. Surf. Sci. 560, 150009. 10.1016/j.apsusc.2021.150009

[B51] Yang W. Yang X. Li B. Lin J. Gao H. Hou C. (2019a). Ultrathin nickel hydroxide nanosheets with a porous structure for efficient electrocatalytic urea oxidation. J. Mat. Chem. A 7 (46), 26364–26370. 10.1039/c9ta06887b

[B52] Zaher A. Shehata N. (2021). Recent advances and challenges in management of urea wastewater: a mini review. IOP Conf. Ser. Mat. Sci. Eng. 1046 (1), 012021. 10.1088/1757-899x/1046/1/012021

[B53] Zhang W. Jia Q. Liang H. Cui L. Wei D. Liu J. (2020). Iron doped Ni_3_S_2_ nanorods directly grown on FeNi_3_ foam as an efficient bifunctional catalyst for overall water splitting. Chem. Eng. J. 396, 125315. 10.1016/j.cej.2020.125315

[B54] Zhang X. Liu Y. Xiong Q. Liu G. Zhao C. Wang G. (2017). Vapour-phase hydrothermal synthesis of Ni_2_P nanocrystallines on carbon fiber cloth for high-efficiency H_2_ production and simultaneous urea decomposition. Electrochim. Acta 254, 44–49. 10.1016/j.electacta.2017.09.097

[B55] Zheng L. Ma S. Wang Z. Shi Y. Zhang Q. Xu X. (2019). Ni-P nanostructures on flexible paper for morphology effect of nonenzymatic electrocatalysis for urea. Electrochim. Acta 320, 134586. 10.1016/j.electacta.2019.134586

[B56] Zhu B. Liang Z. Zou R. (2020). Designing advanced catalysts for energy conversion based on urea oxidation reaction. Small 16 (7), 1906133. 10.1002/smll.201906133 31913584

