# Supplementary material for: Large scale uniform Ni-P plated carbon fiber for boosting urea electro-oxidation and electro-detection
Source: Front Chem. 2023 Oct 26;11:1298655. doi: 10.3389/fchem.2023.1298655 (PMC10639144; doi:10.3389/fchem.2023.1298655)
Supplement: Supplementary file 1 [file DataSheet1.docx]

Supporting Information

**Large Scale Uniform Ni-P Plated Carbon Fiber for Boosting Urea Electro-oxidation** **and Electro-detection**

Yan-ru Fan ^a,^ *, Jin-qi Li ^d^, Yu-xi Yang ^c^, Zhi-hao Zhang ^b^, Jie Zhang ^c^, Jing-he Yang ^d^.

^a^ Clinical Lab department, Henan Provincial People’s Hospital, Zhengzhou,China

^b^ Department of Infections Disease，The First Affiliated Hospital of Zhengzhou University, Zhengzhou 450001, China

^c^ School of Ecology and Environment, Zhengzhou University, Zhengzhou 450001, China

^d^ School of Chemistry Engineering, Zhengzhou University, Zhengzhou 450001, China


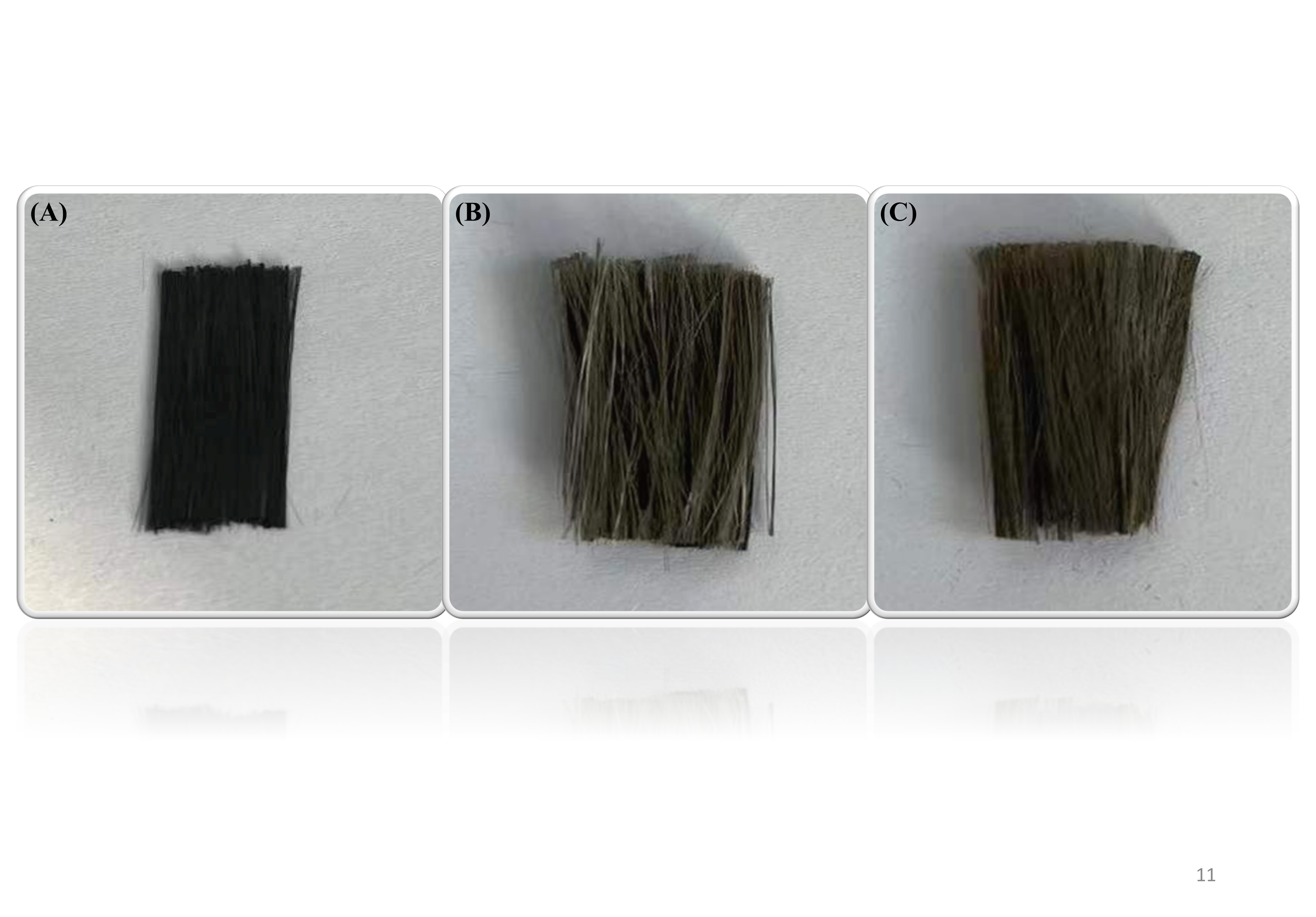


Fig. S1. Images of the corresponding materials. (A) CF; (B) Ni-P/CF.


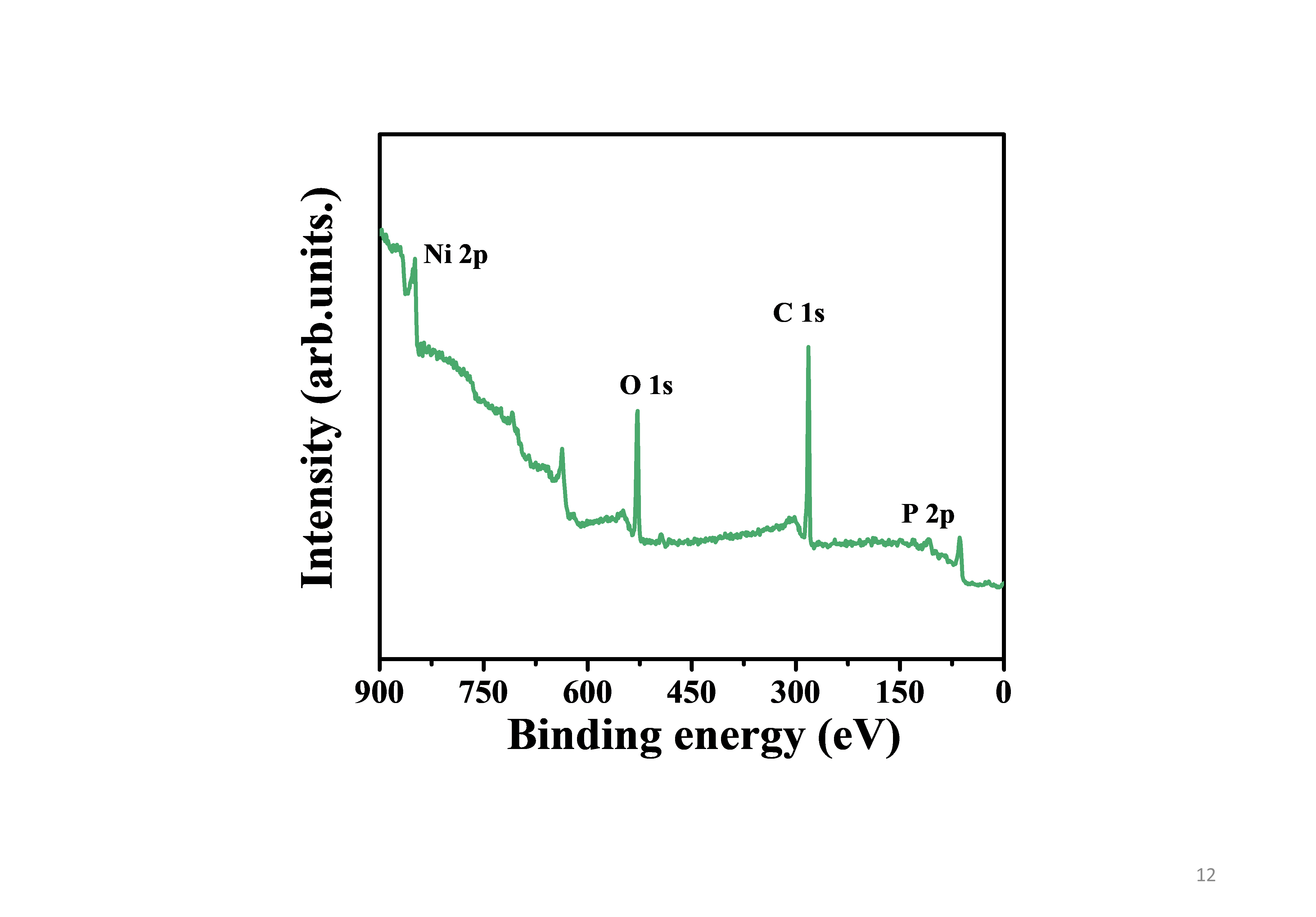


Fig. S2. The XPS survey spectrum of Ni-P/CF.


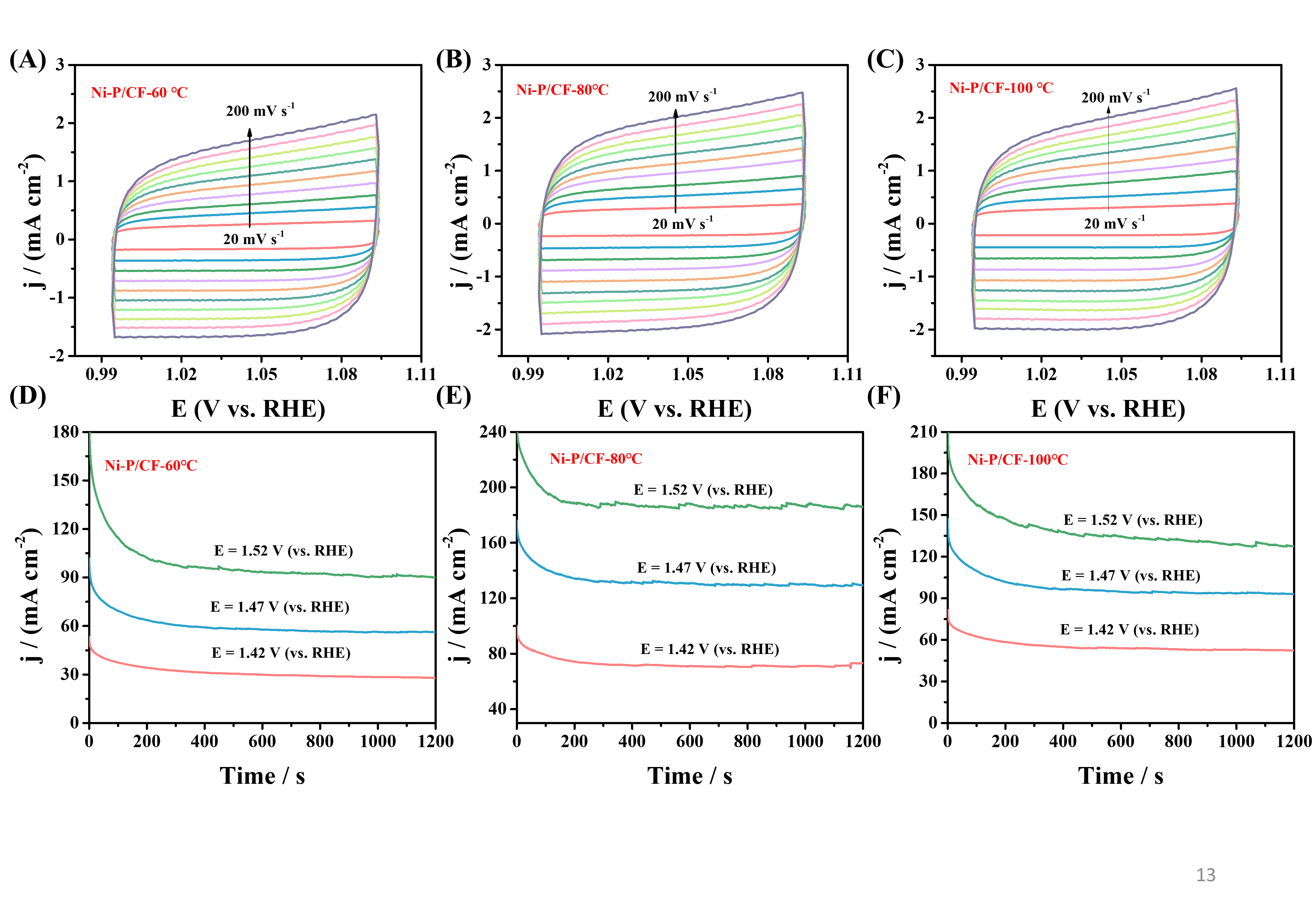


Fig. S3. (A) Cyclic voltammograms at different scan rates from 20 to 200 mV s^-1^ and (B) the I-T curve of Ni-P/CF-60-100℃ at different applied potentials in 1 M KOH with 0.33 M urea.


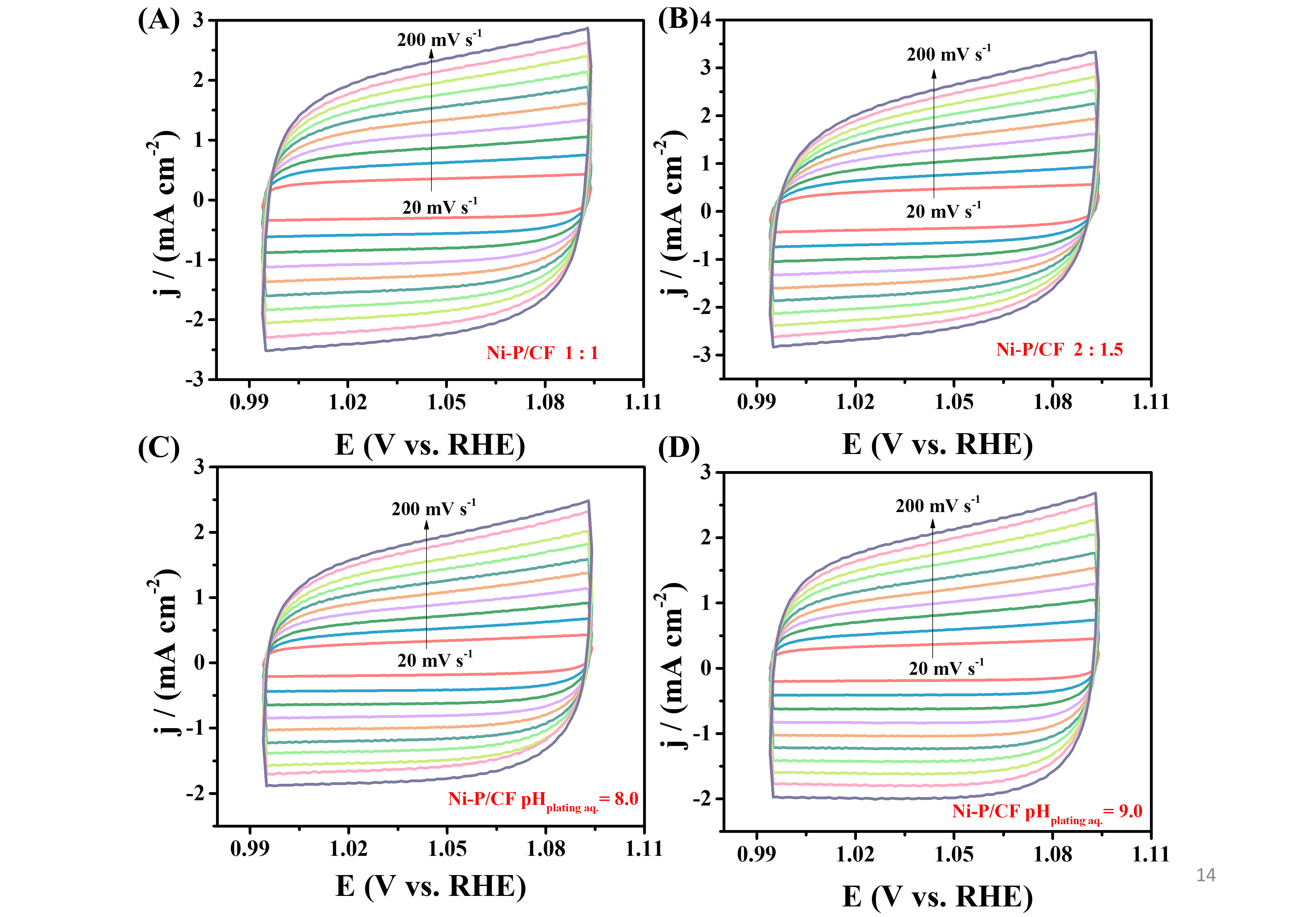


Fig. S4. Cyclic voltammograms of different materials at different scan rates from 20 to 200 mV s^-1^.


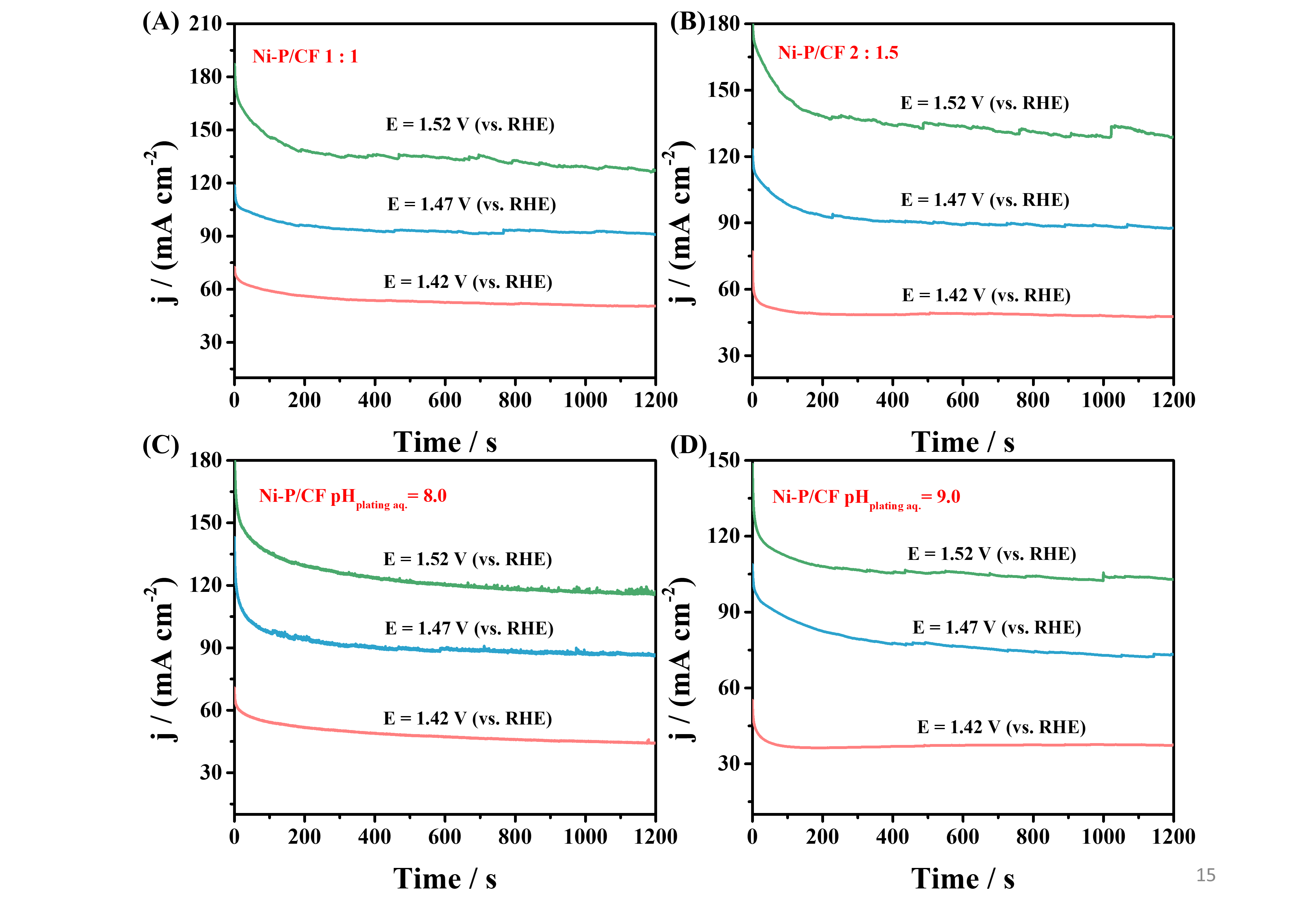


Fig. S5. The I-T curve of different materials at different applied potentials in 1 M KOH with 0.33 M urea, respectively.


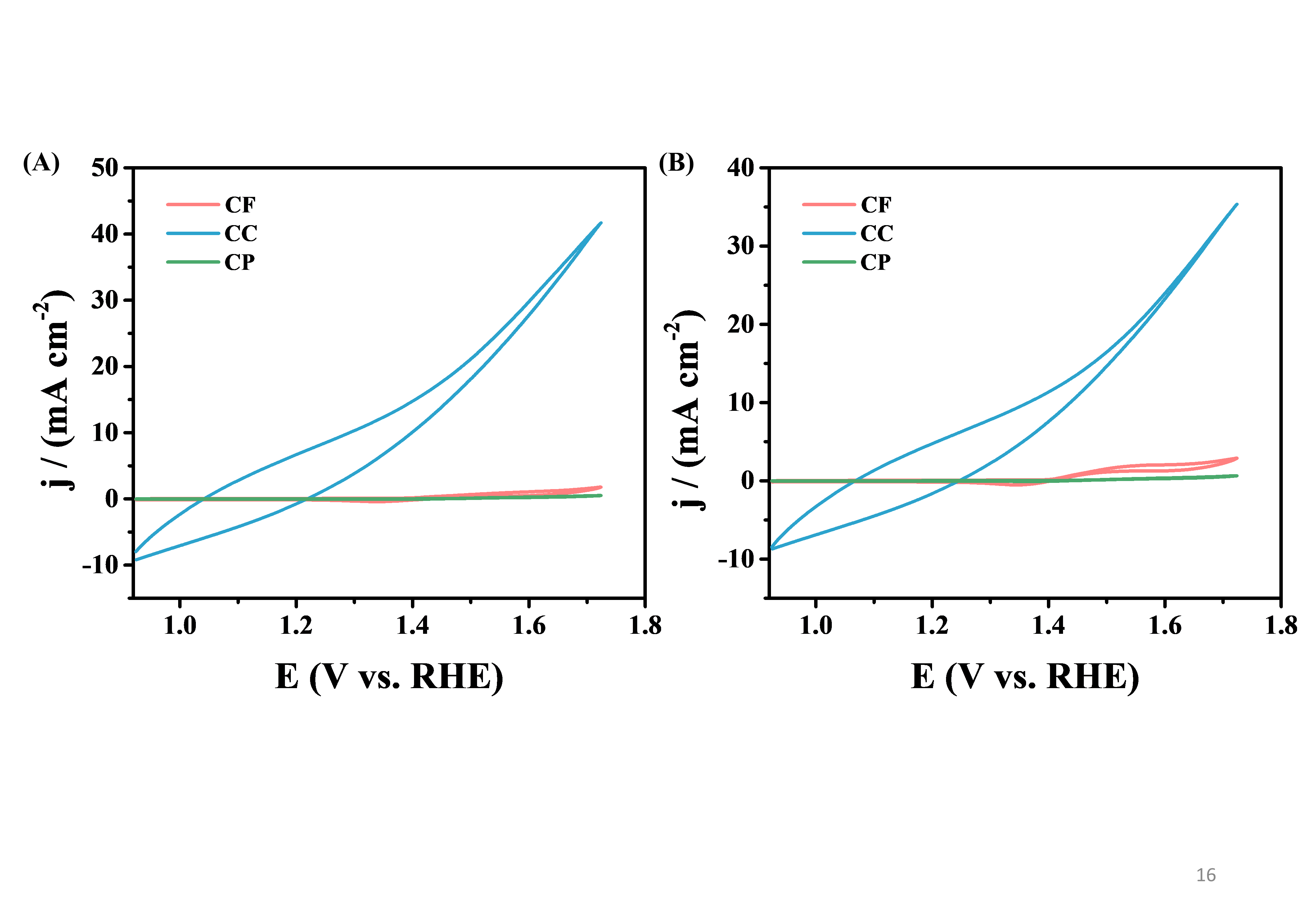


Fig. S6. CVs of these different materials in 1 M KOH without (A) and with (B) 0.33 M urea.


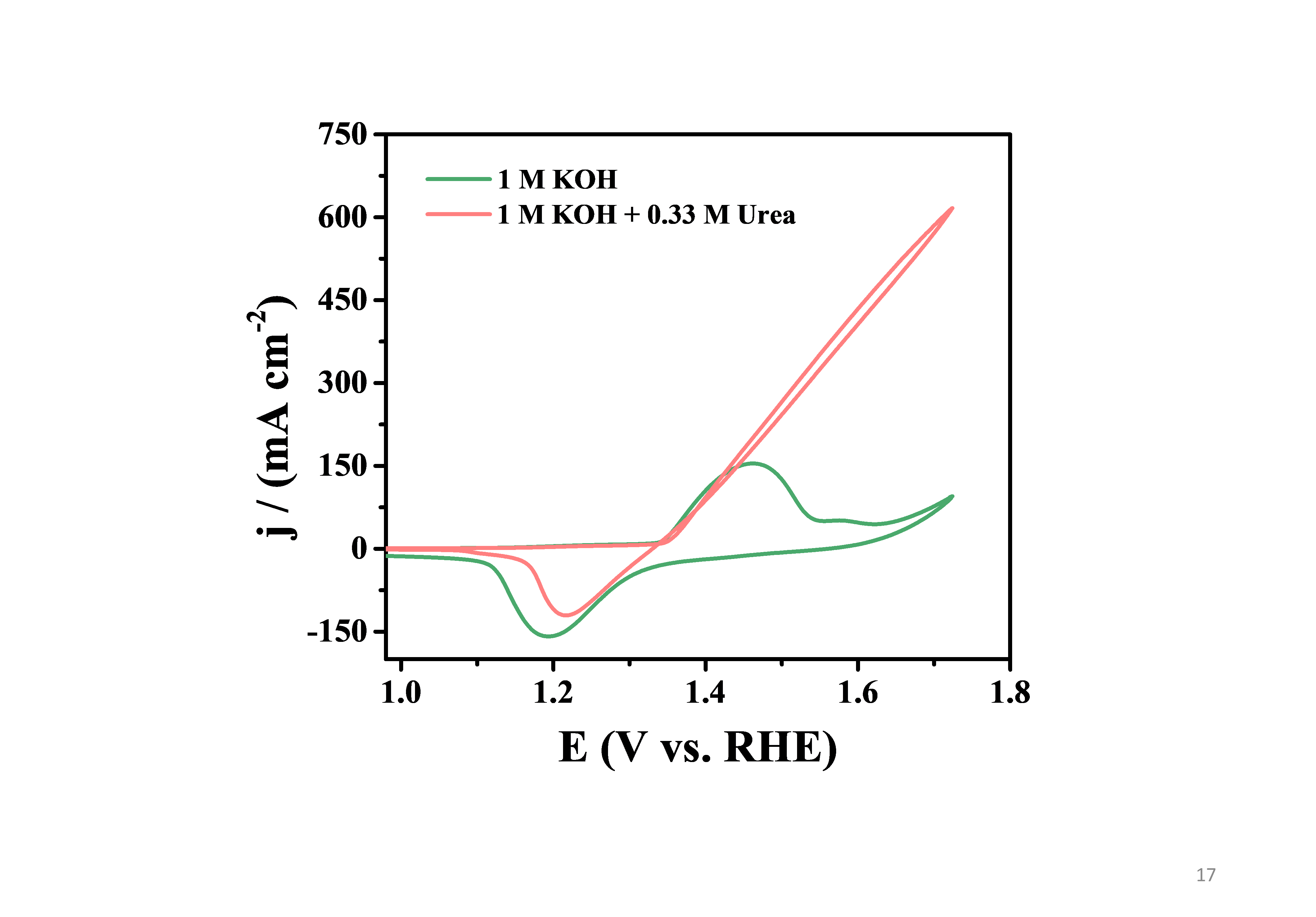


Fig. S7. CVs of Ni-P/CF at 50 mV s^-1^ in 1 M KOH solution with and without 0.33 M urea.


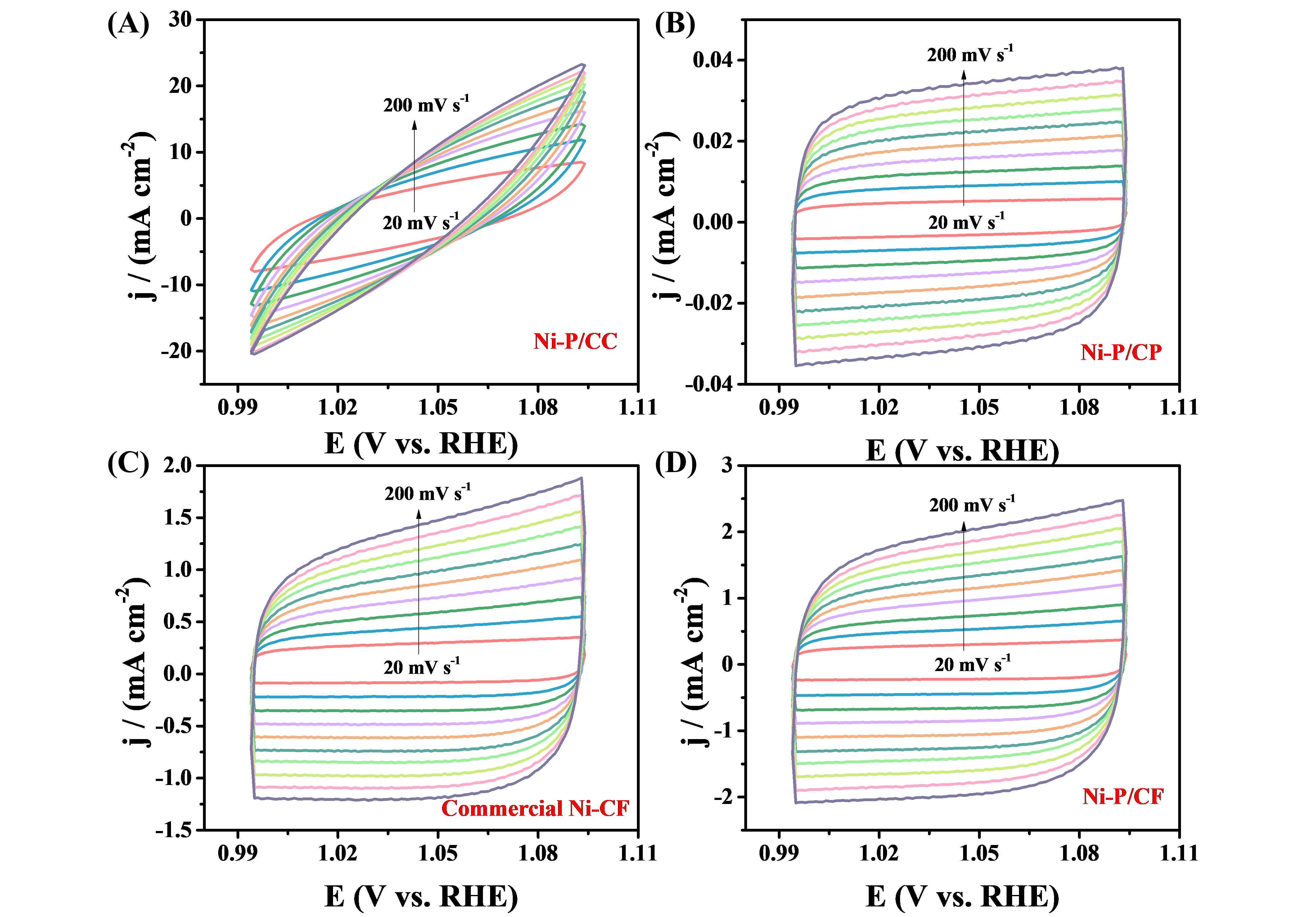


Fig. S8. Cyclic voltammograms of different materials at different scan rates from 20 to 200 mV s^-1^.


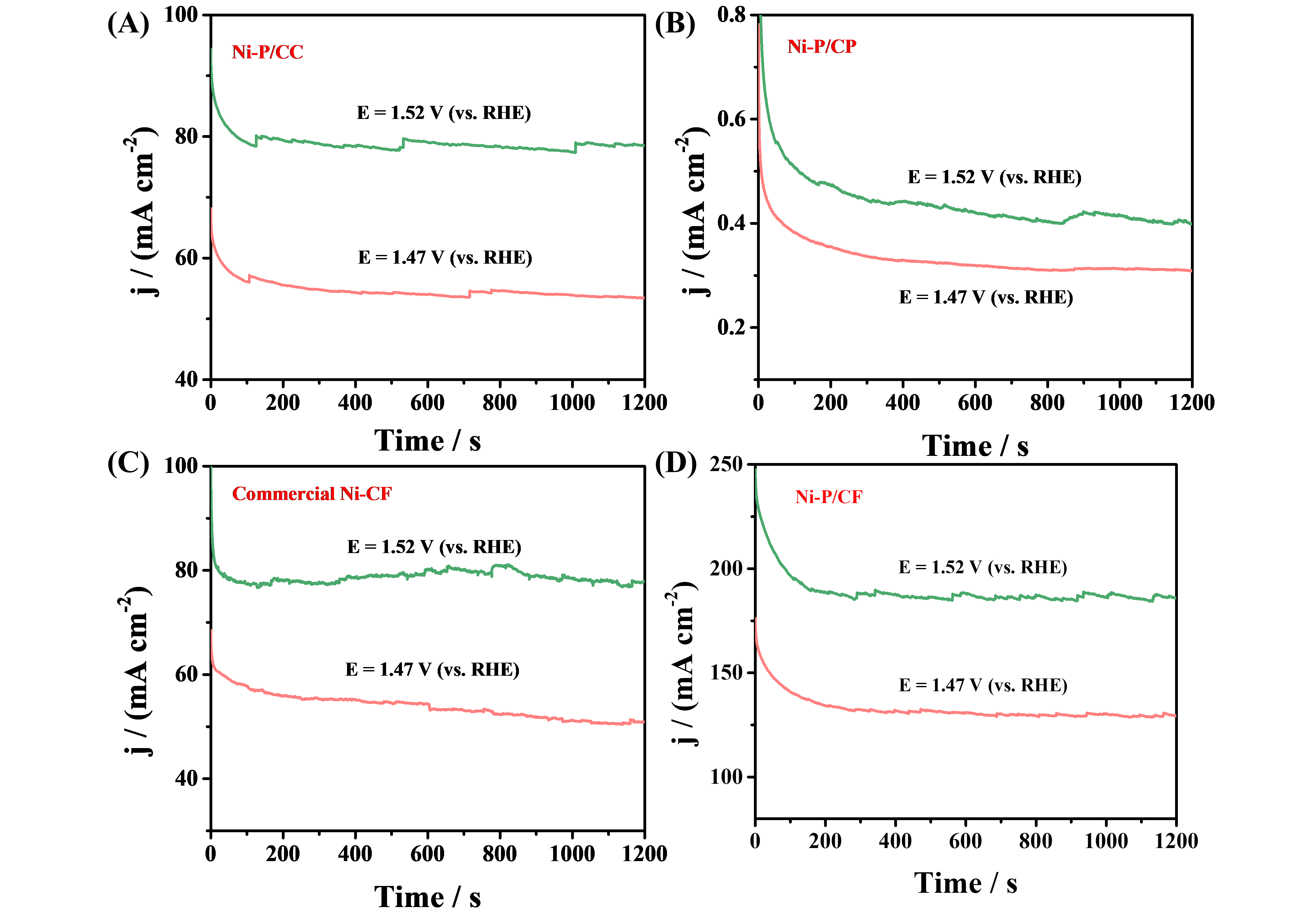


Fig. S9. The I-T curve of different materials at different applied potentials in 1 M KOH with 0.33 M urea, respectively.

Table S1 Electrochemical properties of Ni-P/CF under different process parameters

| Control condition | Parameters | maximum current density  (mA cm^-2^) | R_ct_  (Ω) | C_dl_  (mF cm^-2^) |
| --- | --- | --- | --- | --- |
| Temperature | 60℃ | 380.00 | 2.27 | 16 |
|  | 80℃ | 616.60 | 1.38 | 19 |
|  | 100℃ | 500.20 | 1.95 | 19 |
| Ni/P ratio | 1/1 | 502.20 | 4.86 | 21 |
|  | 1.5/2 | 616.60 | 1.38 | 19 |
|  | 2/1.5 | 460.40 | 11.42 | 23 |
| pH | 8 | 510.80 | 3.89 | 17 |
|  | 8.5 | 616.60 | 1.38 | 19 |
|  | 9 | 496.20 | 5.39 | 19 |

Table S2 Comparison of UOR performance among the reported catalysts

| Catalyst | Solution | | Potential @  j=10 mA cm^-2^  (V vs.RHE) | Ref. |
| --- | --- | --- | --- | --- |
|  | KOH  (M) | Urea  (M) |  |  |
| Ni-P/CF | 1 | 0.33 | 1.34 | This work |
| Ce-Ni_2_P/NF | 1 | 0.3 | 1.41 | [1] |
| NiF_3_/Ni_2_P@CC | 1 | 0.33 | 1.36 | [2] |
| Ni(OH)_2_@NF | 1 | 0.3 | 1.35 | [3] |
| NiFe NSs/NF | 1 | 0.33 | 1.33 | [4] |
| CoFe LDH/MOF | 1 | 0.33 | 1.45 | [5] |
| MOF-Ni@MOF-Fe-S | 1 | 0.5 | 1.35 | [6] |
| Ni_3_N/NF | 1 | 0.5 | 1.34 | [7] |
| Ni_2_P/Fe_2_P/NF | 1 | 0.5 | 1.36 | [8] |
| Fe-Ni_3_S_2_@FeNi_3_-8 | 1 | 0.33 | 1.40 | [9] |
| Co-Ni_3_S_4_NiS/Ni | 1 | 0.5 | 1.35 | [10] |
| NiFe(OH)_x_/Ni_3_N | 1 | 1 | 1.36 | [11] |
| Ni@NCNF-3 | 1 | 0.5 | 1.38 | [12] |
| NiO-NiPi | 1 | 0.5 | 1.35 | [13] |

**REFERENCES**

[1] K. Xiong, L. Yu, Y. Xiang, H. Zhang, J. Chen, Y. Gao, Cerium-incorporated Ni_2_P nanosheets for enhancing hydrogen production from overall water splitting and urea electrolysis, J. Alloys Compd. 912 (2022) 165234.

[2] K. Wang, W. Huang, Q. Cao, Y. Zhao, X. Sun, R. Ding, W. Lin, E. Liu, P. Gao, Engineering NiF_3_/Ni_2_P heterojunction as efficient electrocatalysts for urea oxidation and splitting, Chem. Eng. J. 427 (2022) 130865.

[3] L. Xia, Y. Liao, Y. Qing, H. Xu, Z. Gao, W. Li, Y. Wu, In Situ Growth of Porous Ultrathin Ni(OH)_2_ Nanostructures on Nickel Foam: An Efficient and Durable Catalysts for Urea Electrolysis, ACS Appl. Energ. Mater. 3(3) (2020) 2996-3004.

[4] Y. Diao, Y. Liu, G. Hu, Y. Zhao, Y. Qian, H. Wang, Y. Shi, Z. Li, NiFe nanosheets as urea oxidation reaction electrocatalysts for urea removal and energy-saving hydrogen production, Biosens. Bioelectron. 211 (2022) 114380.

[5] S. Huang, Y. Wu, J. Fu, P. Xin, Q. Zhang, Z. Jin, J. Zhang, Z. Hu, Z. Chen, Hierarchical CoFe LDH/MOF nanorods array with strong coupling effect grown on carbon cloth enables efficient oxidation of water and urea, Nanotechnology 32(38) (2021) 385405.

[6] H. Xu, K. Ye, K. Zhu, J. Yin, J. Yan, G. Wang, D. Cao, Efficient bifunctional catalysts synthesized from three-dimensional Ni/Fe bimetallic organic frameworks for overall urea electrolysis, Dalton Trans 49(17) (2020) 5646-5652.

[7] S. Hu, C. Feng, S. Wang, J. Liu, H. Wu, L. Zhang, J. Zhang, Ni_3_N/NF as Bifunctional Catalysts for Both Hydrogen Generation and Urea Decomposition, ACS Appl Mater Interfaces 11(14) (2019) 13168-13175.

[8] L. Yan, Y. Sun, E. Hu, J. Ning, Y. Zhong, Z. Zhang, Y. Hu, Facile in-situ growth of Ni_2_P/Fe_2_P nanohybrids on Ni foam for highly efficient urea electrolysis, J. Colloid Interface Sci. 541 (2019) 279-286.

[9] W. Zhang, Q. Jia, H. Liang, L. Cui, D. Wei, J. Liu, Iron doped Ni_3_S_2_ nanorods directly grown on FeNi_3_ foam as an efficient bifunctional catalyst for overall water splitting, Chem. Eng. J. 396 (2020) 125315.

[10] Y.-T. Wang, X.-F. He, X.-M. Chen, Y. Zhang, F.-T. Li, Y. Zhou, C. Meng, Laser synthesis of cobalt-doped Ni_3_S_4_-NiS/Ni as high-efficiency supercapacitor electrode and urea oxidation electrocatalyst, Appl. Surf. Sci. 596 (2022) 153600.

[11] H. Zhang, X. Meng, J. Zhang, Y. Huang, Hierarchical NiFe Hydroxide/Ni_3_N Nanosheet-on-Nanosheet Heterostructures for Bifunctional Oxygen Evolution and Urea Oxidation Reactions, ACS Sustain. Chem. Eng. 9(37) (2021) 12584-12590.

[12] Q. Zhang, F.M.D. Kazim, S. Ma, K. Qu, M. Li, Y. Wang, H. Hu, W. Cai, Z. Yang, Nitrogen dopants in nickel nanoparticles embedded carbon nanotubes promote overall urea oxidation, Appl. Catal. B-Environ. 280 (2021) 119436.

[13] X. Xu, T. Guo, J. Xia, B. Zhao, G. Su, H. Wang, M. Huang, A. Toghan, Modulation of the crystalline/amorphous interface engineering on Ni-P-O-based catalysts for boosting urea electrolysis at large current densities, Chem. Eng. J. 425 (2021) 130514.
